# Binge Ethanol Drinking Produces Sexually Divergent and Distinct Changes in Nucleus Accumbens Signaling Cascades and Pathways in Adult C57BL/6J Mice

**DOI:** 10.3389/fgene.2018.00325

**Published:** 2018-09-10

**Authors:** Deborah A. Finn, Joel G. Hashimoto, Debra K. Cozzoli, Melinda L. Helms, Michelle A. Nipper, Moriah N. Kaufman, Kristine M. Wiren, Marina Guizzetti

**Affiliations:** ^1^Department of Behavioral Neuroscience, Oregon Health & Science University, Portland, OR, United States; ^2^Research, VA Portland Health Care System, Portland, OR, United States

**Keywords:** alcohol, qPCR arrays, sex differences, hormone signaling, immune function, neurotransmitter metabolism, C57BL/6J mice

## Abstract

We previously determined that repeated binge ethanol drinking produced sex differences in the regulation of signaling downstream of Group 1 metabotropic glutamate receptors in the nucleus accumbens (NAc) of adult C57BL/6J mice. The purpose of the present study was to characterize RNA expression differences in the NAc of adult male and female C57BL/6J mice following 7 binge ethanol drinking sessions, when compared with controls consuming water. This binge drinking procedure produced high intakes (average >2.2 g/kg/30 min) and blood ethanol concentrations (average >1.3 mg/ml). Mice were euthanized at 24 h after the 7th binge session, and focused qPCR array analysis was employed on NAc tissue to quantify expression levels of 384 genes in a customized Mouse Mood Disorder array, with a focus on glutamatergic signaling (3 arrays/group). We identified significant regulation of 50 genes in male mice and 70 genes in female mice after 7 ethanol binges. Notably, 14 genes were regulated in both males and females, representing common targets to binge ethanol drinking. However, expression of 10 of these 14 genes was strongly dimorphic (e.g., opposite regulation for genes such as *Crhr2*, *Fos*, *Nos1*, and *Star*), and only 4 of the 14 genes were regulated in the same direction (*Drd5*, *Grm4*, *Ranbp9*, and *Reln*). Interestingly, the top 30 regulated genes by binge ethanol drinking for each sex differed markedly in the male and female mice, and this divergent neuroadaptive response in the NAc could result in dysregulation of distinct biological pathways between the sexes. Characterization of the expression differences with Ingenuity Pathway Analysis was used to identify Canonical Pathways, Upstream Regulators, and significant Biological Functions. Expression differences suggested that hormone signaling and immune function were altered by binge drinking in female mice, whereas neurotransmitter metabolism was a central target of binge ethanol drinking in male mice. Thus, these results indicate that the transcriptional response to repeated binge ethanol drinking was strongly influenced by sex, and they emphasize the importance of considering sex in the development of potential pharmacotherapeutic targets for the treatment of alcohol use disorder.

## Introduction

Alcohol use disorder (AUD) is a clinical problem of great significance that cost the United States $249 billion in 2010, with 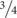 of the cost related to binge drinking or a pattern of drinking that brings blood alcohol concentration ≥ 80 mg/dL (or 0.8 mg/mL; NIAAA, 2004). Excessive alcohol use is the fourth leading preventable cause of death in the United States, but globally, it accounts for 5.9% of all deaths (∼3.3 million in 2012) and is the first leading risk factor for premature death and disability among people between the ages of 15 and 49 (NIAAA, 2017). Epidemiological evidence indicates that women develop alcohol-related heart disease, liver damage, and peripheral neuropathy after fewer years of heavy drinking, and that women may be more vulnerable to AUD-induced brain damage (Wiren, 2013 and references therein).

Behavioral, biochemical, and molecular pharmacological evidence indicates that *N*-methyl-D-aspartate (NMDA) receptors are one of the primary targets of ethanol. Other primary targets include γ-aminobutyric acid_A_ (GABA_A_), glycine, serotonin-3, and neuronal nicotinic acetylcholine receptors, as well as L-type calcium channels and G protein-activated inwardly rectifying potassium channels (reviewed in Spanagel, 2009; Cui and Koob, 2017). Concentrations as low as 1 mM produce alterations in the function of these receptors and ion channels, which initiate a cascade of intracellular events and lead to the acute behavioral effects of ethanol that range from disinhibition to sedation and hypnosis (depending on the dose). Given that practically all neurons in the brain are estimated to possess glutamatergic inputs, glutamatergic neurotransmission is in a position to regulate or influence a diverse array of neuronal processes (see Chandler, 2003; Lau and Zukin, 2007; Bell et al., 2016). A large body of evidence also implicates activity-dependent changes in the efficacy of glutamatergic neurotransmission as a major underlying event in the addicted brain (e.g., reviewed in Chandler, 2003; Tzschentke and Schmidt, 2003; Kauer and Malenka, 2007; Szumlinski et al., 2008a; Kalivas, 2009; Bell et al., 2016). Importantly, we recently found that repeated binge drinking recruited sexually divergent signaling cascades downstream of phosphoinositide 3-kinase (PI3K) in the nucleus accumbens (NAc) in C57BL/6J mice, with significant changes in males and females relatively resistant to these changes (Cozzoli et al., 2016). The functional implication of the changes was confirmed by the demonstration that intra-NAc rapamycin, which inhibits mammalian target of rapamycin (mTOR) in the PI3K signaling cascade, significantly decreased binge ethanol drinking in male but not in female mice (Cozzoli et al., 2016). Taken in conjunction with evidence that rapamycin (see Neasta et al., 2014 and references therein) and newly developed mTOR complex 1 inhibitors (Morisot et al., 2018) significantly reduce high ethanol drinking in male rodents, the results by Cozzoli et al. (2016) highlight sex differences in the influence of binge drinking on signaling cascades downstream of PI3K and presumably, metabotropic Group 1 glutamate receptors (mGluR1).

Neuroadaptive responses to binge ethanol consumption are not limited to effects on neurotransmitter systems. Microarray expression analysis in whole brain or in brain regions such as the medial prefrontal cortex (mPFC), NAc, ventral tegmental area (VTA), and amygdala have found that various models of binge drinking produced changes in expression of genes in male rodents that were involved in some of the following networks: glutamate signaling, BDNF (brain derived neurotrophic factor), synaptic vesicle fusion, synaptic transmission, apoptosis, glucocorticoid receptor (GR) signaling, anti-apoptosis, regulation of G-protein receptor signaling, transcription factors, neurogenesis, and neuroimmune-related pathways (e.g., Rodd et al., 2008; Bell et al., 2009; McBride et al., 2010; Mulligan et al., 2011; Wolstenholme et al., 2011; Agrawal et al., 2014). Binge drinking in female rodents produced changes in VTA gene expression that were associated with neuroimmune and epigenetic functions, a pro-inflammatory response, and an enhanced response to glucocorticoids and steroid hormones (McBride et al., 2013; Marballi et al., 2016), and the two top networks identified were neurological/psychological disorders and lipid/nucleic acid metabolism (Marballi et al., 2016). Changes in NAc and amygdala protein expression in female rats following binge drinking were associated with functional categories such as the cytoskeleton, cellular stress response, membrane transport, and neurotransmission (Bell et al., 2006). Although male and female rodents were never directly compared in the same study, binge-like ethanol drinking changed the expression of genes and proteins that likely alter neuronal function in several ways and that can be either adaptive or deleterious.

Chronic ethanol intoxication that results in physical dependence via continuous or intermittent ethanol vapor exposure also produces gene expression changes, with a different transcriptional response in the cortex during acute withdrawal (8 h) than after a period of abstinence (3 weeks) in male and female rodents. Studies conducted in male mice during acute withdrawal found that transcriptionally responsive genes in the PFC were involved in the Ras/MAPK (mitogen-activated protein kinase) pathway, notch signaling, and ubiquitination (Melendez et al., 2012) and that dysregulation in the expression of several chromatin remodeling genes in PFC was primarily evident during acute withdrawal rather than during a period of abstinence (Hashimoto et al., 2017). Pathways identified in cingulate cortex of male rats after a period of abstinence were involved in neurotransmission (e.g., glutamatergic, endocannabinoid, monoaminergic), signal transduction (e.g., MAPK, ERK2 or extracellular signal-related kinase 2), and synaptic plasticity (Rimondini et al., 2002). Additionally, a study designed to discover master regulator genes (i.e., key genes that drive the expression of the specific transcriptional response associated with physical dependence) during abstinence in male rats identified *Nr3c1*, the gene encoding the GR, as one of the highest master regulators in the mPFC, NAc, VTA, and central nucleus of the amygdala (Repunte-Canonigo et al., 2015). Importantly, several studies were conducted in male and female mPFC with the goal of examining sex and ethanol withdrawal severity genotype differences in gene expression profiles in mice selectively bred for high and low withdrawal. During acute withdrawal, sex rather than withdrawal genotype, correlated best with the transcriptional response in dependent mice. Females showed regulation of genes associated with cell death/neurodegeneration, DNA/RNA binding, and inflammation/immune function whereas males showed regulation of genes associated with protein degradation, calcium ion binding pathways, inflammation/immune function, and nervous system disorders/development (Hashimoto and Wiren, 2008; Wilhelm et al., 2014). However, while NF-κB (nuclear factor kappa-light-chain-enhancer of activated B cells) signaling was identified as a significant signaling node in both males and females, the interacting proteins were completely distinct between the sexes, which was indicative of a sexually dimorphic immune response during acute withdrawal. Subsequent studies during acute withdrawal focused on glucocorticoid signaling, and bioinformatics of genes regulated in dependent mice identified activation of inflammatory signaling and cell death pathways in females, while males exhibited disease and disorder pathways that were associated with endocrine and neurological diseases (Wilhelm et al., 2015). In contrast, abstinence produced a transcriptional response that varied by withdrawal genotype rather than sex. In the low withdrawal genotype, genes associated with the biological processes thyroid hormone metabolism, glutathione metabolism, axonal guidance, and DNA damage response were identified. Classes of genes associated with acetylation and histone deacetylase were highly dimorphic between mice with a high versus (vs.) low withdrawal genotype. The top pathway identified was Death Receptor Signaling, with apoptosis as a central node, but both sexes of the withdrawal resistant genotype had increased apoptotic signaling and more up-regulated transcripts whereas the high withdrawal genotype mice had less apoptotic signaling (Hashimoto et al., 2011; Wilhelm et al., 2014). Collectively, the available data indicate that acute withdrawal following chronic intoxication or binge drinking produces sexually divergent transcriptional responses and activation of distinct networks.

Based on the above evidence for a strong dichotomy between male and female rodents in the response to ethanol during acute withdrawal, the purpose of the present study was to characterize RNA expression differences from male and female C57BL/6J mice following 7 binge ethanol sessions. Tissue was harvested from the NAc, as this brain region is a central mediator of addiction (e.g., Tzschentke and Schmidt, 2003; Kauer and Malenka, 2007; Kalivas, 2009; Koob and Volkow, 2010). Focused quantitative PCR (qPCR) array analysis was employed to quantify expression levels of 384 genes identified as important in “Mood Disorders.” The results indicated that there was a largely divergent regulation of genes by binge drinking in males and females, reflecting different neuroadaptive responses in the NAc that would result in dysregulation of distinct biological pathways between the sexes.

## Materials and Methods

### Subjects

Adult male and female C57BL/6J mice were purchased from Jackson Laboratories West (Sacramento, CA, United States) at 7 weeks of age. Mice were group housed and separated by sex upon arrival, acclimated to a regular 12 h light/dark cycle (lights on at 0700) in a temperature (22 ± 2°C) and humidity controlled environment, with free access to food (Labdiet 5001 rodent chow; PMI International, Richmond, IN, United States) and water. Mice were 8 weeks old at the start of the drinking study, and they were individually housed throughout the experiment. Stage of the estrous cycle was not monitored during this study, based on evidence that binge ethanol consumption was not affected by estrous cycle phase in female C57BL/6J mice and that 6 weeks of binge ethanol drinking did not affect the length or pattern of the estrous cycle (Satta et al., 2018). Results in female rats also indicate that phases of the estrous cycle did not influence ethanol drinking under binge and non-binge drinking conditions (Priddy et al., 2017). The procedures were carried out in accordance with recommendations of the National Institute of Health *Guidelines for the Care and Use of Laboratory Animals* and were compliant with Institutional Animal Care and Use Committee approved protocols. The specific protocol for these studies was approved by the Institutional Animal Care and Use Committee at the VA Portland Health Care System, where all studies were conducted. All efforts were made to minimize distress and the number of animals used.

### Binge Ethanol Consumption

The Scheduled High Alcohol Consumption procedure was used to model binge drinking, based on evidence that this procedure produces high ethanol intake in male and female mice (≥2g/kg in 30 min) and blood ethanol concentrations (BECs) ≥ 1.0 mg/mL (details in Finn et al., 2005; Strong et al., 2010; Tanchuck et al., 2011; Cozzoli et al., 2016). Briefly, mild fluid restriction was used to schedule periods of fluid access so that mice would drink their daily fluid requirement on a schedule. Mice had free access to food, and animals were weighed daily. Total fluid access per day increased across time from 4 to 10 h. Every 3rd day, mice in the binge ethanol groups (binge; 9/sex) had 30 min access to a 5% v/v ethanol solution in tap water, with water provided during the remainder of the period of fluid access. This 3-day cycle of fluid access was repeated so that mice in the binge group received a total of 7 binge ethanol sessions. Retro-orbital sinus blood (20 μL) was collected immediately following the 3rd and 7th binge sessions from the binge groups and analyzed for BEC via headspace gas chromatography (Finn et al., 2007). Mice in the control group (control; 9/sex) received the same schedule of total fluid access, but consumed only water. After the final binge ethanol session, all mice were given free access to water for 24 h.

### Tissue Dissection

Mice were euthanized by decapitation at 24 h after the final binge ethanol session. The brain was extracted, chilled on ice, and sectioned freehand, as described in Cozzoli et al. (2016). Briefly, the entire NAc was micropunched from the 1–2 mm coronal section containing the anterior commissure with a 16 gauge hollow needle, based on established anatomical coordinates from the mouse brain atlas (Paxinos and Franklin, 2001). Micropunches were aimed to include the following coordinates: AP: +1.45 mm from bregma, ML: ± 0.6 mm from the midsagittal suture, DV: -4.3 mm from the skull surface. All samples were placed in microcentrifuge tubes (1.5 ml), frozen immediately in dry ice, and stored at -80°C until total RNA isolation.

### RNA Isolation and Quantitative Polymerase Chain Reaction (qPCR) Array Analysis

Total RNA was isolated using RNA STAT-60 (Tel-Test, Inc.; Friendswood, TX, United States), and genomic DNA was removed with the DNA-Free RNA kit (Zymo Research; Irvine, CA, United States), using routine procedures (e.g., Hashimoto and Wiren, 2008; Hashimoto et al., 2011). First strand cDNA synthesis was carried out on the purified RNA samples (1 μg) with the RT^2^ First Strand Kit (Qiagen, Valencia, CA, United States). Quantitative PCR was performed using customized neuroscience mouse qPCR arrays (Custom 384 Mouse StellARray or Mouse Mood Disorder array) by Bar Harbor BioTechnology (Trenton, ME, United States). A total of 12 qPCR arrays, 384-well PCR plates with primers targeting genes related to Mood Disorders (2.6 ng/reaction), were run by Bar Harbor BioTechnology, with three biological replicates for each sex (male, female) and treatment (binge, control). PCR plates were run on an ABI 7900 HT Real-Time instrument, and data were analyzed with SDS 2.4 software (ABI), using automatic baseline settings with a manual threshold of 0.096 across all samples. For samples with undetectable expression of any gene, a *C*t-value of 40 was assigned to that gene to allow statistical analysis. Quantitative PCR arrays have been documented to provide reliable data that do not require further confirmation, as validation studies in our laboratory have found 100% reproducibility of these data with traditional qPCR methods for testing the expression of individual genes (e.g., Wheeler et al., 2009; Wiren et al., 2010; Wilhelm et al., 2015).

Binge and control mice were chosen for the arrays, based on specific criteria. For binge mice, choices were based on the following: (1) Animals with seven binge sessions > 2 g/kg/30 min or with the greatest number of binges ≥ 2 g/kg were chosen; and (2) Mice with the most consistent BECs that exceeded binge BEC (0.80 mg/mL) were chosen. For the water control mice, choices were based on the following: (1) Consistent 30 min water intake across the 7 “binge” sessions and consistency across animals per group with group average. We also ensured that body weights were not significantly different in the control and binge animals that were chosen.

The qPCR arrays allow for the identification of changes in the expression of pre-selected gene networks associated with specific signaling cascades and pathways that are altered following repeated bouts of binge drinking. We had two rationales for using the Mouse Mood Disorder array. First, the Mouse Mood Disorder array was used by collaborators in our department to examine genes relevant to selection for high and low methamphetamine consumption, given that many of the 384 genes represented on the array are relevant to findings from other studies of addiction related processes (Wheeler et al., 2009). Second, we were able to customize the array to increase the representation of a few glutamatergic genes, including mGluR5, Homer 2, and the PI3K regulatory subunit, which are altered following various models of ethanol drinking in male rodents (e.g., Szumlinski et al., 2008b; Cozzoli et al., 2009, 2016; Obara et al., 2009). Therefore, we wanted to focus this initial examination on a subset of genes most likely to be relevant to addiction (also see Introduction for justification to increase representation of glutamate-related genes). Additionally, several advantages to the qPCR arrays exist, such as: (a) The use of qPCR arrays does not require the confirmation of gene expression differences as is required for more comprehensive arrays (e.g., Affymetrix), since qPCR is the usual confirmation procedure; and (b) We have considerable expertise in the use of qPCR arrays (e.g., Wheeler et al., 2009; Wiren et al., 2010; Wilhelm et al., 2015) and corresponding bioinformatics (Hashimoto and Wiren, 2008; Hashimoto et al., 2011, 2017; Wilhelm et al., 2014, 2015) to identify expression differences.

### Quantitative Reverse-Transcriptase PCR (qRT-PCR)

Using NAc tissue from a separate group of binge and control mice, we performed real time qRT-PCR to examine the expression of additional genes not present on the array but that were implicated in downstream signaling cascades of pathways that were identified by Ingenuity Pathway Analysis (IPA) of the current qPCR array data as being affected by binge ethanol drinking (*n* = 4/sex/treatment). Real time qRT-PCR was performed with the iCycler IQ Real Time PCR detection system (Bio-Rad Laboratories, Inc., Hercules, CA, United States), using a one-step QuantiTect SYBR Green RT-PCR kit (Qiagen) on DNase-treated total RNA (Hashimoto et al., 2004). The qRT-PCR reactions were carried out in 25 μL with 20 ng of total RNA that was isolated from mice that were not used in the qPCR arrays. Primers were purchased pre-designed from Qiagen.

Real-time qRT-PCR efficiency was determined for each primer set by using a fivefold dilution series of total RNA, and it did not differ significantly from 100%. Specificity of the qPCR reaction was confirmed with melt curve analysis to ensure that only the expected PCR product was amplified. Relative expression of the qRT-PCR product was determined using the comparative ΔΔ*C*t method, after normalizing expression to total RNA measured with RiboGreen (Molecular Probes, Eugene, OR, United States; Hashimoto et al., 2004).

### Statistical Analyses

Data were analyzed using R or SYSTAT (version 11, SYSTAT Software, Inc., Richmond, CA, United States). The level of significance was set at *p* ≤ 0.05, and *p* ≤ 0.09 was considered a trend. Results are presented as mean ± SEM.

For the drinking data, the dependent variables were BEC, volume (in mLs) of water and ethanol consumed, ethanol dose consumed (in g/kg), and body weight. Analysis of variance (ANOVA) was used to assess day, sex (male, female) and treatment (binge, control) effects or binge day and sex effects when only the ethanol data were examined. Significant interactions were followed up with *post hoc* tests. Because we were predicting sex differences, planned comparisons were conducted with or without the presence of a significant interaction.

For the qPCR array data, Bar Harbor BioTechnology identified significantly changed genes in the data set using their Global Pattern Recognition (GPR) algorithm (Akilesh et al., 2003). GPR goes through several iterations to compare the expression of each gene to every other gene in the array, establishing a global pattern where significant changes are identified and ranked. The procedure looks for the most stably expressed genes from the array across all the samples, and uses these genes to normalize the gene expression. Akilesh et al. (2003) validated that GPR provided a novel alternative to the use of relative normalization in qPCR experiments and emphasized that GPR takes advantage of biological replicates to obtain significant changes in gene expression. For comparative purposes, *p*-values from the GPR analysis were then used to calculate *q*-values to control for multiple comparisons using the “qvalue” package in R (version 2.12.0^1^). However, we used an uncorrected *p*-value to decrease the chance of excluding regulated transcripts (i.e., false negatives) as we (Hashimoto and Wiren, 2008; Hashimoto et al., 2011; Wilhelm et al., 2014) and others (e.g., see Rodd et al., 2007) have employed. All significantly regulated transcripts (*p* ≤ 0.05) from either comparison (i.e., male binge vs. control, female binge vs. control) were then used to create heat maps and hierarchical clustering using the R package “gplots” (version 3.0.1) with complete linkage clustering. Bioinformatic analyses were conducted by uploading the significantly regulated genes to the IPA website^2^. Proprietary IPA software was used for the analyses, and significance was based on the relative enrichment of the regulated genes to biological function, pathway, or network using the 384 genes present in the qPCR array as the background gene-set and the Fisher’s exact test. Thus, for all the pathway analyses (IPA), using the 384 genes present on the qPCR array as the background gene set controlled for the enrichment of specific gene classes (i.e., related to Mood Disorders) in our set of regulated genes.

For the qRT-PCR data, all data were calculated as fold change relative to the female controls after normalizing expression to total RNA measured with RiboGreen. Initial analyses were conducted with ANOVA to assess sex and treatment effects. When there was a significant interaction, *post hoc t*-tests were conducted to examine treatment effects in each sex.

## Results

### Binge Drinking

**FIGURE 1:**
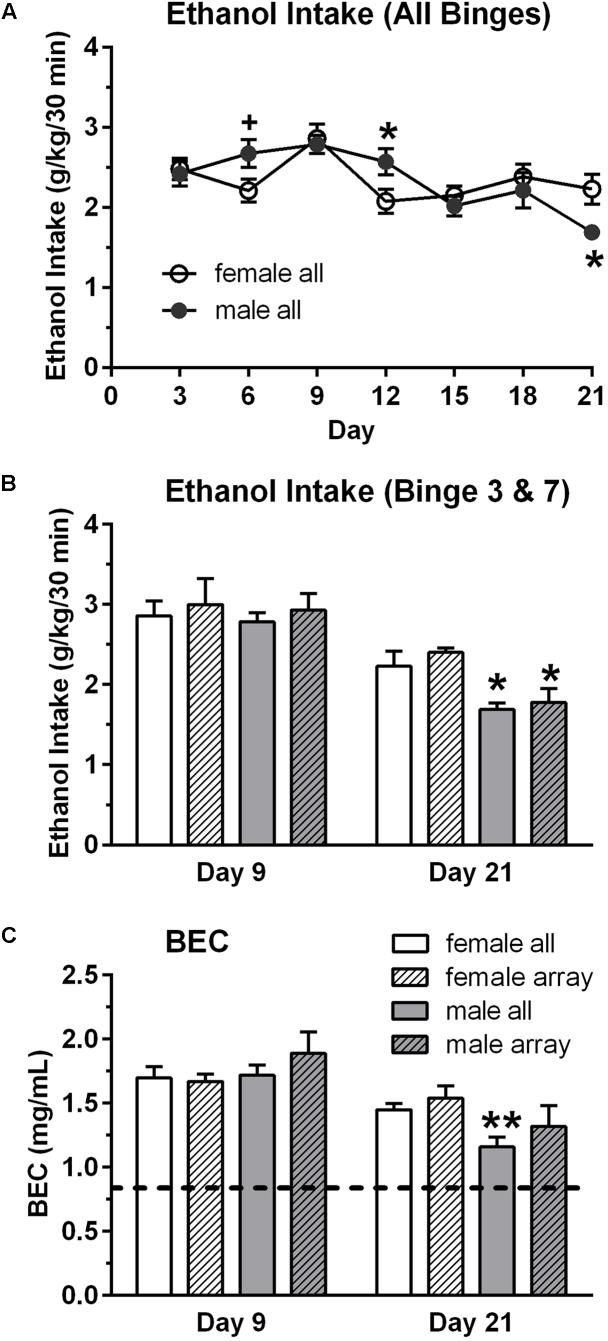
Binge ethanol intake **(A,B)** and blood ethanol concentration (BEC, **C**) in male and female mice. Mice in the binge groups had a total of seven binge drinking sessions, with a binge session every 3rd day **(A)**. BEC was measured at the end of the 3rd (day 9) and 7th (day 21) binge sessions. Although overall binge ethanol intake, averaged across the seven sessions, did not differ in the female (2.34 g/kg) and male (2.35 g/kg) mice, ethanol intake and corresponding BECs were lower in the male vs. female mice on the final binge session (day 21, **B,C**). However, BECs greatly exceed the criteria for binge drinking on all days (0.80 mg/mL; depicted by dashed line in **C**). Shown are mean ± SEM for all mice in the binge groups (*n* = 9/sex), which included the mice in the subgroup that were used for the qRT-PCR analysis (*n* = 4/sex), and for the mice in the subgroup that were used for the qPCR arrays (*n* = 3/sex). ^+^*p* = 0.06, ^∗^*p* < 0.05, ^∗∗^*p* < 0.01 vs. respective female all or female array group.

Male and female mice had seven intermittent binge ethanol sessions (binge, 9/sex) or consumed water (control, 9/sex). Overall binge ethanol intake (g/kg/30 min) did not differ between the sexes, when collapsed across the seven binge sessions. Mean ± SEM intake was 2.34 ± 0.06 g/kg for females and 2.35 ± 0.10 g/kg for males. However, analysis of the seven binge ethanol sessions revealed that the pattern of ethanol intake across time differed in the male vs. female mice [time: *F*(6,84) = 7.02, *p* < 0.001; sex × time: *F*(6,84) = 2.61, *p* < 0.05]. Ethanol intake was significantly lower in female vs. male mice on day 12 (4th binge, *p* < 0.05, **Figure 1A**) and was significantly higher in female vs. male mice on day 21 (7th binge, *p* < 0.05, **Figures 1A,B**). The slight decrease in ethanol intake across binge sessions in the male mice likely reflects the increase in fluid access time across sessions, which is a finding that we have observed in some of our prior studies using this binge drinking procedure. BECs were measured after the 3rd (day 9) and 7th (day 21) binge ethanol sessions (**Figure 1C**), and they mirrored the ethanol intake data [time: *F*(1,16) = 28.28, *p* < 0.001; sex × time: *F*(1,16) = 4.18, *p* = 0.058], with *post hoc* tests confirming that BEC was significantly higher in female vs. male mice on day 21 (*p* < 0.01). A similar pattern of results was found for the subgroup of mice that were chosen for the array analysis (**Figures 1B,C**). Ethanol intake and BECs did not differ in male vs. female mice on day 9, whereas ethanol intake was significantly higher in female vs. male mice on day 21. BEC on day 21 also was higher in females vs. males, but this difference did not reach statistical significance. Importantly, the results confirm that both male and female mice in the binge groups consumed high doses of ethanol in the 30 min binge sessions and achieved BECs that exceeded the NIAAA criteria for binge drinking (0.80 mg/mL; shown as dashed line on **Figure 1C**; NIAAA, 2004).

Body weights, averaged over the 21 days of the study, were lower in female vs. male mice [*F*(1,32) = 364.20, *p* < 0.001]. Averaged body weights also were lower in the control vs. binge groups [*F*(1,32) = 5.59, *p* < 0.05], and this effect was primarily due to the significant difference in the male mice (**Table 1**). Body weights on day 1 of the study were slightly lower in the control mice when compared to the mice in the binge groups, which likely contributed to the significant difference in average body weight. However, weight gain across the 21 days of the study was similar in the control and binge groups for the male (10.1% for binge, 12.0% for control) and female (11.7% for binge, 10.1% for control) mice. Overall total fluid intake did not differ in the control vs. binge groups for the male and female mice (**Table 1**). Thus, treatment (binge vs. control) did not significantly alter body weight gain or total fluid intake in either sex.

**Table 1:** Body weight and total fluid intake during the Scheduled High Alcohol Consumption procedure.

Sex	Treatment	Body weight (g)	Total fluid intake (mL)
Male	Binge	21.59 ± 0.32	3.19 ± 0.11
	Control	20.69 ± 0.20^∗^	3.24 ± 0.12
Female	Binge	16.19 ± 0.26	3.03 ± 0.12
	Control	15.81 ± 0.29	3.20 ± 0.14


***Shown are the mean ± SEM body weights and total fluid intake, averaged over the 21 days of the study, for n = 9/sex and treatment. ^∗^p < 0.05 vs. respective binge.***

### Binge Drinking Produces Sexually Divergent Changes in Gene Expression Patterns Associated With Discrete Biological Pathways

Focused qPCR array analysis was employed to quantify expression levels of 384 genes identified as important in “Mood Disorders” (see **Supplementary Table S3** for genes in array). We found that of the 384 genes on the array, only 14 genes were regulated by binge drinking in both males and females (**Table 2**), representing common targets to binge ethanol consumption. However, only 4 genes were regulated in the same direction (*Drd5*, *Grm4*, *Ranbp9*, and *Reln*), while the expression of 10 genes was strongly dimorphic (*Crhr2*, *Dgka*, *Fos*, *Lta*, *Mc5r*, *Nos1ap*, *Nos1*, *Slc6a2*, *Star*, and *Smc4*) such that the direction of change differed between male and female mice. Additionally, we identified significant regulation by binge drinking of 70 genes in females (**Supplementary Table S1**), and the 30 most highly regulated transcripts in females are shown in **Table 3**. In male mice, a total of 50 genes were regulated significantly by binge ethanol drinking (**Supplementary Table S2**), and the 30 most highly regulated transcripts are shown in **Table 4**.

**Table 2:** Nucleus accumbens genes significantly regulated by binge ethanol drinking in both female and male mice.

Gene symbol	Gene name	Female			Male		
			
		Fold change	*p-*value	*q-*value	Fold change	*p-*value	*q-*value
*Crhr2*	Corticotropin releasing hormone receptor 2	-3.13	0.020	0.066	2.07	0.022	0.064
*Dgka*	Diacylglycerol kinase, alpha	11.02	0.032	0.070	-1.84	0.034	0.065
*Drd5*	Dopamine receptor D5	-2.07	0.034	0.070	-2.30	0.021	0.064
*Fos*	FBJ osteosarcoma oncogene	-3.44	0.004	0.054	1.64	0.030	0.065
*Grm4*	Glutamate receptor, metabotropic 4	-2.22	0.030	0.070	-2.19	0.019	0.064
*Lta*	Lymphotoxin A	-14.33	0.001	0.022	1.88	0.027	0.064
*Mc5r*	Melanocortin 5 receptor	-3.64	0.003	0.051	1.93	0.024	0.064
*Nos1ap*	Nitric oxide synthase 1 (neuronal) adaptor protein	-3.91	0.010	0.064	1.43	0.043	0.066
*Nos1*	Nitric oxide synthase 1, neuronal	2.78	0.042	0.075	-1.91	0.023	0.064
*Ranbp9*	RAN binding protein 9	2.30	0.036	0.070	1.59	0.047	0.066
*Reln*	Reelin	-1.95	0.017	0.064	-2.17	0.011	0.064
*Slc6a2*	Solute carrier family 6 (neurotransmitter transporter, noradrenalin), member 2	-21.58	0.019	0.064	10.49	0.000	0.016
*Star*	Steroidogenic acute regulatory protein	1.90	0.042	0.075	-2.18	0.018	0.064
*Smc4*	Structural maintenance of chromosomes 4	93.99	0.000	0.018	-2.48	0.017	0.064


*A total of 14 genes in the nucleus accumbens (core and shell) were significantly regulated by 7 binge ethanol drinking sessions in male and female mice, but only 4 of the 14 genes were regulated in the same direction in the sexes. Significance is based on p-values, but q-values also are shown. Transcripts are listed in alphabetical order. Fold change of binge vs. control is shown, with negative values indicating down-regulation by binge ethanol drinking and positive values indicating up-regulation by ethanol. We note that 3 of the genes with high fold changes (Smc4 and Dgka in females, Slc6a2 in males and females) had 2 or more samples with undetected expression, indicating qualitative regulation (i.e., present in binge ethanol samples but absent in controls).*

**Table 3:** Top 30 genes significantly regulated by binge drinking in female nucleus accumbens.

Gene symbol	Gene name	Female		
		
		Fold change	*p-*value	*q-*value
*Casp8*	Caspase 8	114.71	0.001	0.021
*Smc4*	Structural maintenance of chromosomes 4	93.99	0.000	0.018
*Alox12*	Arachidonate 12-lipoxygenase	73.14	0.000	0.018
*Pmch*	Pro-melanin-concentrating hormone	28.46	0.001	0.022
*Dgka*	Diacylglycerol kinase, alpha	11.02	0.032	0.070
*Prkcq*	Protein kinase C, theta	9.22	0.035	0.070
*Timeless*	Timeless circadian clock 1	5.33	0.048	0.081
*Esr1*	Estrogen receptor 1 (alpha)	5.00	0.019	0.064
*Dlx1*	Distal-less homeobox 1	4.41	0.002	0.039
*Eif2s2*	Eukaryotic translation initiation factor 2, subunit 2 (beta)	4.07	0.013	0.064
*Hdac1*	Histone deacetylase 1	3.63	0.012	0.064
*Egfr*	Epidermal growth factor receptor	3.50	0.013	0.064
*Pafah1b1*	Platelet-activating factor acetylhydrolase, isoform 1b, subunit 1	2.99	0.015	0.064
*Katnal1*	Katanin p60 subunit A-like 1	2.98	0.023	0.070
*Nos1*	Nitric oxide synthase 1, neuronal	2.78	0.042	0.075
*Fos*	FBJ osteosarcoma oncogene	-3.44	0.004	0.054
*Impa2*	Inositol (myo)-1(or 4)-monophosphatase 2	-3.53	0.035	0.070
*Drd3*	Dopamine receptor D3	-3.53	0.028	0.070
*Mc5r*	Melanocortin 5 receptor	-3.64	0.003	0.051
*Nos1ap*	Nitric oxide synthase 1 (neuronal) adaptor protein	-3.91	0.010	0.064
*St8sia2*	ST8 alpha-N-acetyl-neuraminide alpha-2,8-sialyltransferase 2	-4.08	0.011	0.064
*Nfkbib*	Nuclear factor of kappa light polypeptide gene enhancer in B-cells inhibitor, beta	-4.80	0.012	0.064
*Ppp1r1b*	Protein phosphatase 1, regulatory (inhibitor) subunit 1B	-4.89	0.037	0.070
*Il2rb*	Interleukin 2 receptor, beta chain	-5.14	0.012	0.064
*Il9r*	Interleukin 9 receptor	-6.18	0.007	0.064
*Drd4*	Dopamine receptor D4	-9.72	0.006	0.064
*Prf1*	Perforin 1 (pore forming protein)	-11.61	0.009	0.064
*Lta*	Lymphotoxin A	-14.33	0.001	0.022
*Fosl1*	Fos-like antigen 1	-15.10	0.039	0.073
*Slc6a2*	Solute carrier family 6 (neurotransmitter transporter, noradrenalin), member 2	-21.58	0.019	0.064


*The 15 most highly up- or down-regulated transcripts are shown from the total of 70 genes that were significantly regulated by binge ethanol drinking in females. Significance is based on p-values, but q-values also are shown. Transcripts are listed in ascending order. Fold change of binge vs. control is shown, with positive values indicating up-regulation and negative values indicating down-regulation by ethanol. Some of the genes with high fold changes (Casp8, Smc4, Alox12, Pmch, Dgka, Prkcq, Timeless, Fosl1, and Slc6a2) had 2 or more samples with undetected expression, indicating qualitative regulation (i.e., present in binge, absent in control).*

**Table 4:** Top 30 genes significantly regulated by binge drinking in male nucleus accumbens.

Gene symbol	Gene name	Male		
		
		Fold change	*p-*value	*q-*value
*Fasl*	Fas ligand (TNF superfamily, member 6)	20.15	0.016	0.064
*Chrna1*	Cholinergic receptor, nicotinic, alpha polypeptide 1 (muscle)	15.31	0.019	0.064
*Slc6a2*	Solute carrier family 6 (neurotransmitter transporter, noradrenalin), member 2	10.49	0.000	0.016
*Crh*	Corticotropin releasing hormone	5.32	0.041	0.066
*Ptgs2*	Prostaglandin-endoperoxide synthase 2	4.66	0.009	0.064
*Bdnf*	Brain derived neurotrophic factor	4.26	0.008	0.064
*Crhr2*	Corticotropin releasing hormone receptor 2	2.07	0.022	0.064
*Tuba8*	Tubulin, alpha 8	1.96	0.016	0.064
*Mc5r*	Melanocortin 5 receptor	1.93	0.024	0.064
*Cpt2*	Carnitine palmitoyltransferase 2	1.91	0.025	0.064
*Lta*	Lymphotoxin A	1.88	0.027	0.064
*Dusp6*	Dual specificity phosphatase 6	1.86	0.023	0.064
*Homer2*	Homer homolog 2 (Drosophila)	1.83	0.027	0.064
*Slc6a3*	Solute carrier family 6 (neurotransmitter transporter, dopamine), member 3	1.80	0.028	0.064
*Gabra1*	Gamma-aminobutyric acid (GABA) A receptor, subunit alpha 1	1.67	0.045	0.066
*Grm4*	Glutamate receptor, metabotropic 4	-2.19	0.019	0.064
*Npas2*	Neuronal PAS domain protein 2	-2.20	0.011	0.064
*Drd5*	Dopamine receptor D5	-2.30	0.021	0.064
*Rap1gap*	Rap1 GTPase-activating protein	-2.37	0.033	0.065
*Ccl3*	Chemokine (C-C motif) ligand 3	-2.40	0.049	0.066
*Smc4*	Structural maintenance of chromosomes 4	-2.48	0.017	0.064
*Bcr*	Breakpoint cluster region	-2.52	0.030	0.065
*Htr1b*	5-hydroxytryptamine (serotonin) receptor 1B	-2.80	0.022	0.064
*Isyna1*	Myo-inositol 1-phosphate synthase A1	-2.88	0.015	0.064
*Ace*	Angiotensin I converting enzyme (peptidyl-dipeptidase A) 1	-3.19	0.023	0.064
*Bax*	BCL2-associated X protein	-3.47	0.032	0.065
*Drd1*	Dopamine receptor D1	-3.51	0.011	0.064
*Cit*	Citron	-3.96	0.010	0.064
*Tfap2b*	Transcription factor AP-2 beta	-15.01	0.000	0.013
*S100a9*	S100 calcium binding protein A9 (calgranulin B)	-24.14	0.026	0.064


*The 15 most highly up- or down-regulated transcripts by binge drinking in males are shown. Significance is based on p-values, but q-values also are shown. Transcripts are listed in ascending order. Fold change of binge vs. control is shown, with positive values indicating up-regulation and negative values indicating down-regulation by ethanol. Some of the genes with high fold changes (Fasl, Chrna1, Slc6a2, and S100a9) had 2 or more samples with undetected expression, indicating qualitative regulation (i.e., present in binge, absent in control).*

**FIGURE 2:**
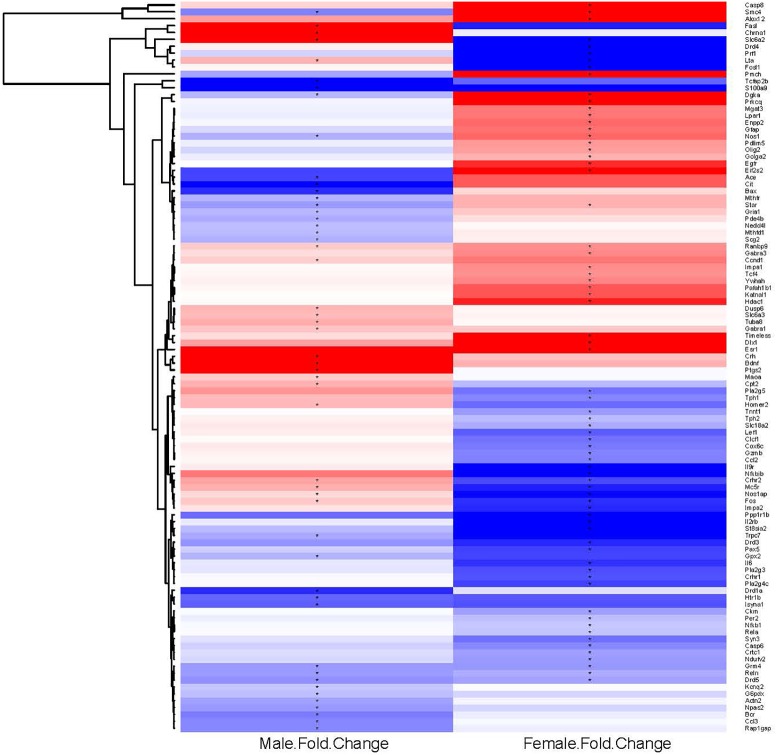
Heat map and hierarchical cluster analysis of genes significantly regulated by repeated binge drinking in male and female mice. All significantly regulated genes (106 total) were used to generate the heat map and to perform the cluster analysis to visualize the transcriptional response at 24 h after the 7th binge ethanol drinking session. Each column represents the combined data from six arrays (three binge, three control), with the binge ethanol-induced change in expression shown for males and females in separate columns. Shades of color indicate up-regulation (red) or down-regulation (blue) for a particular gene following binge drinking. Hierarchical cluster analysis was performed on the ethanol regulated genes. Genes (represented by rows, names at the right of the image) were clustered according to the similarity of expression profile as the result of repeated binge drinking. Clustering of genes emphasizes the sexually dimorphic response to repeated binge drinking experience. ^∗^*p* < 0.05 for significantly regulated genes (binge vs. control).

To examine further the patterns of expression differences, false color heat maps were generated for the transcripts that were regulated significantly by binge drinking, and unsupervised hierarchical cluster analysis was performed to determine the similarity of global expression patterns in the significantly regulated genes by binge ethanol drinking in male and female mice (**Figure 2**). Each column represents the combined data from 6 arrays for each sex (3 binge, 3 control) to visualize the transcriptional response at 24 h withdrawal after the 7th binge ethanol (or water) drinking session. All of the 106 significantly regulated genes were included in this analysis (14 common genes, 56 genes only in females, 36 genes only in males); these genes are depicted based on the GPR fold change for binge ethanol vs. control, with shades of color to indicate up-regulation (red) or down-regulation (blue) for a particular gene following binge ethanol drinking. Clustering analysis was used to identify groups of genes that demonstrated similar expression profiles. Genes (represented by rows in **Figure 2**) were clustered according to the similarity of their expression profile as a result of repeated binge drinking. The gene tree at the left of the image in **Figure 2** corresponds to the degree of similarity in the expression pattern for the specific genes. In general, gene clustering showed the sexually dimorphic response to repeated binge drinking experience.

To better characterize the expression differences observed following repeated binge drinking, IPA was used to identify Canonical Pathways, Upstream Regulators, and significant Biological Functions of regulated genes compared to the background 384 genes present on the qPCR array platform. In female mice, expression differences suggested that hormone signaling and immune function might be altered. Canonical Pathways that were significantly regulated included “Crosstalk between dendritic cells and natural killer cells,” “MIF (macrophage migration inhibitory factor) regulation of innate immunity,” “TNFR1 (tumor necrosis factor receptor 1) signaling,” “TNFR2 signaling,” and “MIF-mediated glucocorticoid regulation” (all *p* < 0.05). Upstream Regulator analysis identified several regulators of expression, such as: POMC (pro-opiomelanocortin), *Tac1* (encodes the protein substance P), *Notch1*, and *Vegf* (all *p* < 0.005). The two top networks included “Neurological Disease, Psychological Disorders, Behavior,” and “Carbohydrate Metabolism, Lipid Metabolism, Small Molecule Biochemistry.” Relationships between regulated genes in these combined networks identified ERK1/2 and Akt (a serine/threonine kinase typically activated by PI3K) as central nodes. Biological Function analysis identified “Infectious Disease” (*p* < 0.005) and “Neurological Disease” (*p* < 0.05) as top targets.

A different pattern of results was found for males, where expression differences suggested that neurotransmitter metabolism was altered by repeated binge drinking. The top Canonical Pathways that were significantly regulated included “nNOS (neuronal nitric oxide synthase) signaling”, “cAMP-mediated signaling,” and “Folate transformations I” (all *p* < 0.05), with a trend for regulation of “Corticotropin releasing hormone (CRH) signaling” (*p* = 0.09). Several upstream regulators of gene expression were identified: indomethacin, apomorphine, Histone h3, and corticosterone (all *p* < 0.0007). The two top networks included “Behavior, Nucleic Acid Metabolism, Small Molecule Biochemistry” and “Psychological Disorders, Neurological Disease, Cell-To-Cell Signaling and Interaction.” Relationships between regulated genes in these combined networks identified PKC (protein kinase C), BDNF, and NMDA as central nodes. Biological Function analysis identified “Neurological Disease” as a top target category (*p* < 0.05).

As suggested by the above pathway analysis, several neurotransmitter systems were influenced by repeated binge drinking sessions. Binge drinking produced an overall suppression in the expression of dopamine receptor genes (**Figure 4D**, bottom 4 genes; see **Supplementary Tables S1**, **S2** for significant binge vs. control gene expression changes in females and males, respectively), with similar fold decreases in expression of *Drd5* (**Table 2**, *p* < 0.05 for both sexes) and *Drd3* in males (*p* < 0.07) and females (*p* < 0.05). Binge drinking also produced a non-significant decrease in expression of *Drd2* in males and females (not shown). However, expression of *Drd1* was only decreased by binge drinking in males (*p* = 0.01), whereas *Drd4* expression was only decreased in females (*p* < 0.01). Binge ethanol drinking also significantly decreased expression of the gene encoding the dopamine transporter (*Slc6a3*) in males (*p* < 0.05) and the gene encoding the vesicular monoamine transporter 2 (*Slc18a2*) in females (*p* < 0.05). However, the gene encoding the norepinephrine transporter (*Slc6a2*) was differentially altered by binge drinking (**Table 2**), where expression was decreased in females (*p* < 0.05) and increased in males (*p* < 0.001). Overall, the functional implication of these binge ethanol-induced changes would likely be a decrease in dopamine signaling in the NAc.

Binge drinking also produced an overall increase in expression of the 4 GABA_A_ receptor subunit genes that were on the arrays in both sexes, with significant changes for 2 of the subunit genes. Expression of *Gabra3* was increased similarly by binge drinking in females (*p* < 0.05) and males (*p* = 0.051), and the similar fold increase in *Gabra1* expression in both sexes only was significant in males (*p* < 0.05). The ethanol-induced increase in *Gabra5* expression only approached the level of a statistical trend for females (*p* = 0.10), whereas *Gabrg2* expression was not significantly altered in either females or males (not shown). Taken in conjunction with the understanding that there are many additional GABA_A_ receptor subunits that can influence GABA_A_ receptor-mediated inhibition, the results are suggestive of a binge ethanol-induced increase in GABA_A_ receptor signaling.

The expression of some glutamatergic genes encoding specific metabotropic and ionotropic receptors also was influenced by binge drinking. With the exception of a similar significant decrease in expression of *Grm4* (**Table 2**), there were differential effects of binge drinking in males and females on the expression of the glutamatergic genes examined (**Figure 4D**, discussed in more detail in section “Pathways Identified by Analysis of Genes That Were Regulated by Binge Drinking in Both Males and Females”).

### Pathways Identified by Analysis of Genes That Were Regulated by Binge Drinking in Both Males and Females

**FIGURE 3:**
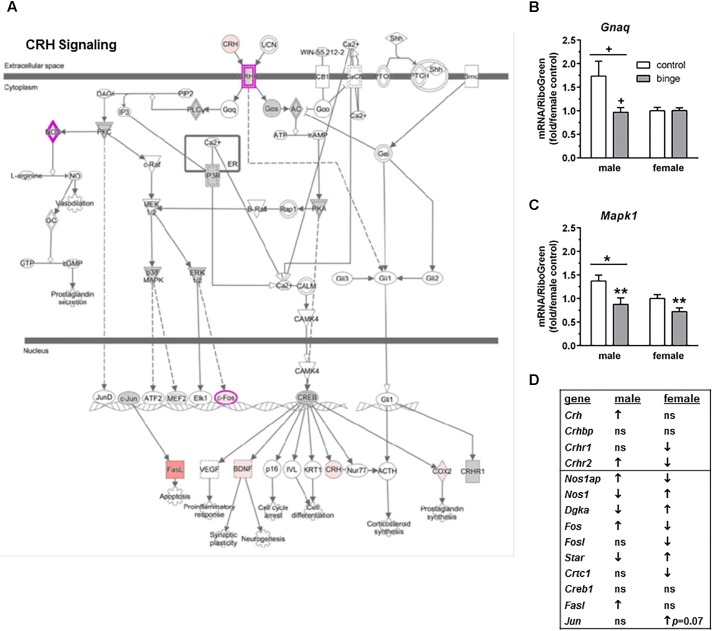
Simplified corticotropin releasing hormone (CRH) signaling pathway highlights genes influenced by repeated binge drinking in male and female mice. This canonical pathway was identified by IPA as regulated by binge drinking in both males and females. **(A)** Depicts the CRH signaling pathway and highlights genes regulated by binge drinking in males (pink for up-regulation, green for down-regulation). **(B,C)** Depict qRT-PCR results and show that expression of *Gnaq* (**B** which encodes Gαq) tended to be higher in males vs. females and to be decreased by binge ethanol drinking in males. Expression of *Mapk1* (**C** which encodes Erk2) was significantly higher in males vs. females and was significantly decreased by binge drinking in both sexes. Values are the mean ± SEM for 4/sex/treatment. ^+^*p* < 0.07 vs. respective control in males or sex difference (over horizontal line); ^∗^*p* < 0.05 for main effect of sex (over horizontal line), ^∗∗^*p* < 0.01 for main effect of treatment. **(D)** Shows significant regulation by binge drinking of select genes from the qPCR array analysis that are pertinent to the CRH signaling cascade depicted in **(A)** for male and female mice (↑ for up-regulation, ↓ for down regulation; *p* < 0.05 at a minimum). For statistical trends, the *p*-values are provided. *Gnaq* (guanine nucleotide binding protein, alpha q polypeptide) encodes the protein Gαq. *Mapk1* (mitogen-activated protein kinase 1) encodes the protein ERK2 (extracellular signal-regulated kinase).

We ran an IPA of genes that were regulated by binge drinking in both males and females and identified three canonical pathways of interest. For each pathway, we identified genes that had the potential to be significantly altered by ethanol and then used Real-Time qRT-PCR to examine the expression of those transcripts. The first pathway identified was “CRH signaling” (**Figure 3A** highlights changes in expression in males). Interestingly and as shown in **Figure 3D** (top 4 genes on table), females show inactivation of the pathway (↓ in *Crhr1* and *Crhr2*), while males show activation of the pathway (↑ in *Crh* and *Crhr2*). We conducted qRT-PCR on *Gnaq* (which encodes Gαq), *Mapk1* (mitogen-activated protein kinase, which encodes Erk2) and *Mapk3* (which encodes Erk1). Expression of *Gnaq* tended to be lower in females vs. males (main effect of sex, *p* < 0.07), with a significant interaction between sex and treatment (*p* < 0.05). *Post hoc* tests showed that mRNA levels tended to be decreased by binge drinking in males (*p* = 0.06; **Figure 3B**). The result in males is consistent with the identification of PKC as a central node, suggesting that signaling downstream following binding to CRH receptors does occur via Gαq in males. In females, it is likely that signaling downstream of CRH receptors favors Gαs. For both sexes, qPCR array results indicate that binge drinking up-regulated *Gnas* (encodes Gαs, guanine nucleotide binding protein, alpha stimulating) by 1.70-fold in females (*p* < 0.14) and 1.34-fold in males (*p* = 0.11), but these differences vs. control were not statistically significant. Expression of *Mapk1* (**Figure 3C**), but not *Mapk3* (not shown), was significantly lower in females vs. males (main effect of sex, *p* < 0.05) and was significantly decreased by binge drinking in both sexes (main effect of treatment, *p* < 0.01). ERK1/2 had been identified as a central node in females, so the binge drinking-induced decrease in *Mapk1* expression in females would be consistent with the decreased expression of *Fos* following binge drinking in this sex, as it is a downstream target of ERK1/2 (**Figures 3A,D**).

**FIGURE 4:**
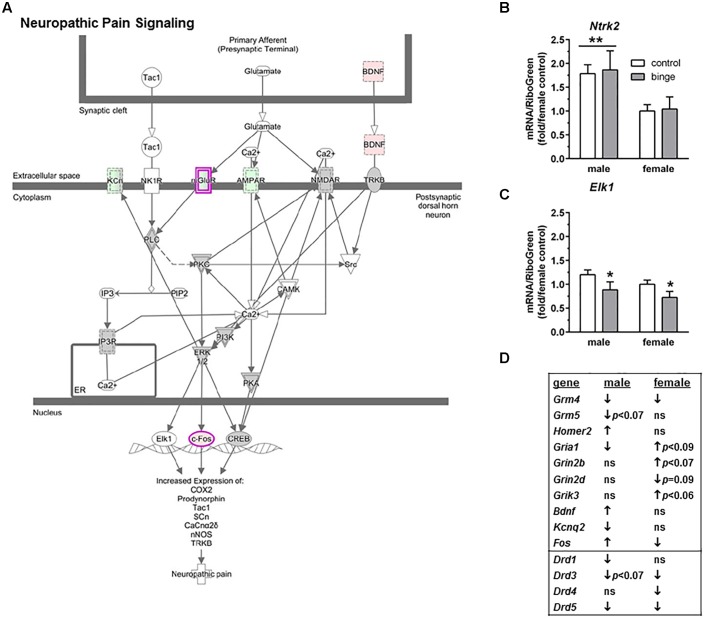
Simplified neuropathic pain signaling pathway highlights genes influenced by repeated binge drinking in male and female mice. This canonical pathway was identified by IPA as regulated by binge drinking in both males and females. **(A)** Depicts the neuropathic pain signaling pathway and highlights genes regulated by binge drinking in males (pink for up-regulation, green for down-regulation). **(B,C)** Depict qRT-PCR results and show that expression of *Ntrk2* (**B** which encodes TrkB) was significantly higher in males than in females. Expression of *Elk1* (**C** which encodes transcription factor Elk1) was significantly decreased by binge drinking in both sexes. Values are the mean ± SEM for 4/sex/treatment. ^∗^*p* < 0.05 for main effect of treatment, ^∗∗^*p* = 0.01 for main effect of sex (over horizontal line). **(D)** Shows significant regulation by binge drinking of select genes from the qPCR array analysis in male and female mice that are pertinent to the neuropathic signaling cascade depicted in **(A)** (top 10 genes) or that are pertinent to effects on other receptor systems (↑ for up-regulation, ↓ for down regulation; *p* ≤ 0.05 at a minimum). For statistical trends, the *p*-values are provided. *Ntrk2* (neurotrophic tyrosine kinase, receptor, type 2) encodes the protein TrkB (tropomyosin receptor kinase B). *Elk1* (ELK1, member of ETS oncogene family) encodes the transcription factor Elk1.

The second pathway identified was “Neuropathic pain signaling” (**Figure 4A** highlights changes in expression seen in males). As shown in **Figure 4D** (top 10 genes on table), binge drinking produced a more complex change in the expression of genes in the neuropathic pain pathway, which was focused on glutamatergic and BDNF signaling. In males, binge drinking produced a significant increase in the expression of BDNF and a significant decrease in expression of AMPA receptors and mGluR4. Expression of mGluR5 tended to be decreased by binge drinking in males, while expression of Homer2 was significantly increased. In females, binge drinking produced a similar significant decrease in *Grm4* (**Table 2** and **Figure 4D**) but opposite effects on the remaining glutamatergic genes and BDNF. For the qRT-PCR analysis, we chose *Ntrk2* (which encodes TrkB, tropomyosin receptor kinase B), *Elk1* (which encodes the transcription factor Elk1), *Mapk1* and *Mapk3* as follow-up candidates. We also were interested in *Creb1*, but qPCR array analysis showed that the expression of this gene was not significantly altered by binge drinking in either sex (not shown). Expression of *Ntrk2* was significantly lower in females vs. males (main effect of sex, *p* = 0.01), but there was no effect of binge drinking (**Figure 4B**). However, *Elk1* expression was significantly decreased by binge drinking in both males and females (main effect of treatment, *p* < 0.05). As mentioned above, expression of *Mapk1* (**Figure 3C**) also was significantly decreased by binge drinking in males and females. At least in females, the binge drinking-induced decrease in expression of *Mapk1* (encoding for Erk2) corresponds with the decreased expression of the transcription factors *Elk1* (**Figure 4C**) and *Fos* (**Figure 4D**), which likely influence downstream gene expression mediated by these transcription factors.

**FIGURE 5:**
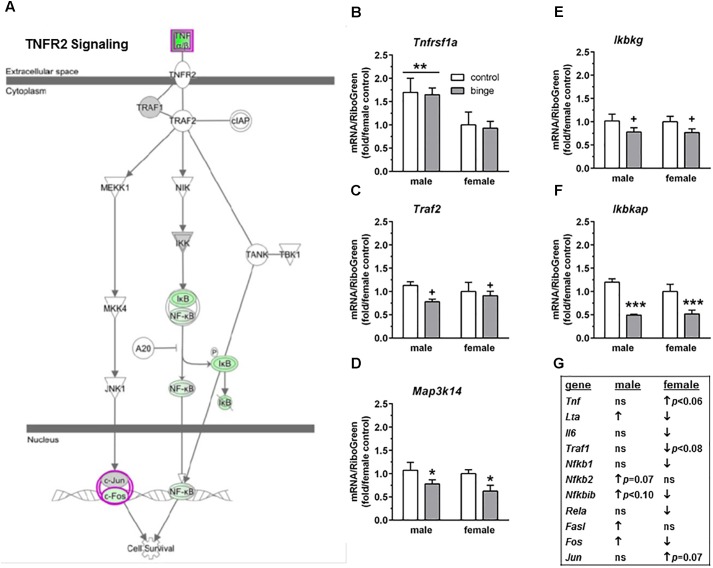
Simplified tumor necrosis factor receptor 2 (TNFR2) signaling pathway highlights genes influenced by repeated binge drinking in male and female mice. This canonical pathway was identified by IPA as regulated by binge drinking in both males and females. **(A)** Depicts the TNFR2 signaling pathway and highlights genes regulated by binge drinking in females (pink for up-regulation, green for down-regulation). **(B–F)** Depict qRT-PCR results. Expression of *Tnfrsf1a* (**B** which encodes TNFR1 and forms a heterocomplex with TNFR2) was significantly higher in males vs. females. However, *Map3k14* (**D** encodes NIK) and *Ikbkap* (**F** encodes IKAP) expression was significantly decreased by binge drinking in both males and females, whereas expression of *Traf2* (**C** encodes TRAF2) and *Ikbkg* (**E** encodes IKK-γ) trended toward a decrease by binge drinking in both sexes. Values are the mean ± SEM for 4/sex/treatment. ^+^*p* < 0.09, ^∗^*p* < 0.05, ^∗∗∗^*p* < 0.001 for main effect of treatment, ^∗∗^*p* < 0.01 for main effect of sex (over horizontal line). **(G)** Shows significant regulation by binge drinking of select genes from the qPCR array analysis in male and female mice that are pertinent to the TNFR2 signaling cascade depicted in **(A)** (↑ for up-regulation, ↓ for down regulation; *p* < 0.05 at a minimum). For statistical trends, the *p*-values are provided. *Tnfrsf1a* (tumor necrosis factor receptor superfamily, member 1a) encodes TNFR1, which is a member of the TNF receptor superfamily of proteins. *Traf2* (TNF receptor-associated factor 2) encodes TRAF2. *Map3k14* (mitogen-activated protein kinase kinase kinase 14) encodes NIK. *Ikbkg* (inhibitor of kappaB kinase gamma) encodes IKK-γ or NEMO, which is one of three subunits that forms the IκB kinase (IKK) enzyme complex. *Ikbkap* (inhibitor of kappa light polypeptide gene enhancer in B-cells, kinase complex-associated protein) encodes IKAP.

The third pathway identified was “TNFR2 signaling” (**Figure 5A** highlights changes in expression seen in females). Interestingly and as shown in **Figure 5G**, the females show inactivation of the pathway (↓ in *Nfkb1*, *Nfkbib*, *Fos*, *Lta*, *Il6*, and *Rela*), while males show activation of the pathway (↑ in *Fos*, *Fasl*, and *Lta*, with a trend for ↑ in *Nfkb2* and *Nfkbib*). We conducted qRT-PCR on several genes in this signaling cascade: *Tnfrsf1a* (encodes TNFR1, which forms a heterocomplex with TNFR2; both receptors bind TNFα), *Mapk8* (encodes JNK1), *Traf2* (encodes TRAF2), *Map3k14* (encodes NIK), and 3 genes encoding subunits in the IκB kinase enzyme complex [*Chuk* (encodes IKK-α or IKK1), *Ikbkb* (encodes IKK-β or IKK2), and *Ikbkg* (encodes IKK-γ or NEMO)]. We also examined *Ikbkap*, which encodes a protein (IKAP) that was initially thought to be a scaffolding protein for the IκB kinase complex. Expression of *Tnfrsf1a* was significantly lower in females vs. males (main effect of sex, *p* < 0.01), but there was no effect of binge drinking (**Figure 5B**). Expression of *Chuk* and *Ikbkb* also was not altered by binge drinking in either sex (not shown). However, *Map3k14* (**Figure 5D**) and *Ikbkap* (**Figure 5F**) expression was significantly decreased by binge drinking in both males and females (main effect of treatment, *p* < 0.05 and *p* < 0.001, respectively). Expression of *Traf2* (**Figure 5C**) and *Ikbkg* (**Figure 5E**) tended to be decreased by binge drinking in both sexes (main effect of treatment, *p* < 0.09 and *p* = 0.06, respectively). Additionally, the gene *Lta*, which encodes the protein lymphotoxin-alpha or TNF-β, was differentially altered by binge drinking (**Table 2** and **Figure 5G**), where expression was decreased in females (*p* = 0.001) and increased in males (*p* < 0.05). In general, the results in females demonstrate that binge drinking produces a fairly consistent downregulation of signaling through the tumor necrosis factor (TNF) superfamily, which likely influences activation of the transcription factor NF-κB.

## Discussion

The present results add to a body of evidence indicating that binge drinking and chronic ethanol intoxication leading to the development of physical dependence both produce neuroadaptive changes in neurotransmitter systems as well as numerous other cellular pathways that can alter neuronal function in a manner that can be either adaptive or deleterious (see section “Introduction”). Importantly, because we directly tested males and females following repeated binge drinking, the present results show for the first time that repeated binge drinking experience produces sexually divergent transcriptional responses and activation of distinct networks, similar to what has been reported for males and females tested during acute withdrawal following chronic intoxication (Hashimoto and Wiren, 2008; Wilhelm et al., 2014, 2015). Of the 106 genes significantly affected by binge drinking in the present study, only 4 were regulated similarly in males and females, demonstrating a profound sex difference in neuroadaptive responses in the NAc that would result in dysregulation of distinct biological pathways between the sexes. For instance, IPA identified Psychological Disorders and Neurological Disease as one of the top two networks, based on the expression differences following repeated binge drinking in male and female mice. However, the relationships between genes identified distinct molecules as significant signaling nodes, suggestive of a sexually dimorphic response that also may be related to mood disorders.

It was not surprising that neurotransmission was significantly affected by binge drinking. The current results are consistent with prior microarray studies that identified networks or biological processes related to glutamate signaling, BDNF and synaptic transmission in the NAc and central nucleus of the amygdala (Rodd et al., 2008; McBride et al., 2010) or in the PFC (Wolstenholme et al., 2011) from male rodents following binge drinking and networks related to neurotransmission in the NAc and amygdala from female rats following binge drinking (Bell et al., 2006). Neurotransmission also was one pathway identified in the cingulate cortex of dependent male rats after a period of abstinence, which included the glutamatergic and monoaminergic systems (Rimondini et al., 2002). Based on the results in dependent male rats during abstinence, in conjunction with the binge drinking-related changes in expression of glutamatergic and dopaminergic genes in male mice in the present study, it is possible that repeated binge drinking experience produces neuroadaptive changes in glutamatergic and dopaminergic signaling that continue through the development of dependence and a period of abstinence, at least in male rodents. Consistent with this idea, 3 months of chronic ethanol intake produced a significant increase in NAc Homer2 protein levels that persisted at 2 months of abstinence in male C57BL/6J mice, and Homer2 overexpression in the NAc facilitated the effect of single or repeated ethanol injections on extracellular glutamate and dopamine levels in the NAc (Szumlinski et al., 2008b). Likewise, NAc Homer2 and mGluR5 protein levels were significantly elevated at 1 month of abstinence after 6 months of chronic ethanol drinking (Obara et al., 2009). Collectively, a large body of evidence indicates that changes in glutamate receptors, transporters, enzymes, and scaffolding proteins are critical for the development of dependence and addiction (see reviews by Szumlinski et al., 2008a; Kalivas, 2009; Bell et al., 2016).

Earlier work with the Scheduled High Alcohol Consumption model of binge drinking found that repeated bouts of binge drinking increased NAc protein levels of Homer2, NMDA receptor 2A and 2B subunits, and PI3K activation in male C57BL/6J mice, without altering protein levels of mGluR1 and mGluR5 at 24 h after the final binge session (Cozzoli et al., 2009). Recently, we replicated the lack of effect of binge drinking on NAc protein levels of mGluR1 and mGluR5, but we also observed an ethanol-induced decrease in RNA expression of *Grm1* and *Grm5* in male C57BL/6J mice, and comparable changes were not found in female C57BL/6J mice (Cozzoli et al., 2016). In the present study, binge drinking significantly increased *Homer2* expression and tended to decrease *Grm5* expression only in male mice (*Grm1* was not on the arrays), and there was a non-significant increase in *Pik3r1* expression in female (↑ 1.48-fold, *p* = 0.12) and male (↑ 1.17-fold, *p* < 0.20) mice. As we discuss in Cozzoli et al. (2016), it is possible that some of the differences between studies were due to whether the experiments were conducted in the circadian dark vs. light phase. The majority of studies that observed a binge drinking-induced activation of PI3K at 24 h of abstinence were conducted during the circadian dark phase (Cozzoli et al., 2009, 2012; Neasta et al., 2010 – but see Neasta et al., 2011), whereas binge ethanol drinking occurred during the circadian light phase in the current and our recent studies (Cozzoli et al., 2016). In the studies by Cozzoli et al. (2016), 24 h of abstinence following repeated binge drinking significantly decreased activation of PI3K and mammalian target of rapamycin (mTOR) protein levels in NAc tissue from male but not female mice. It is interesting that intra-NAc administration of rapamycin to inhibit mTOR signaling significantly decreased binge drinking in male but not female mice (Cozzoli et al., 2016). This result suggested that rapamycin blocked a binge ethanol-induced activation of mTOR in males, because it was administered prior to the binge ethanol session, and that the ethanol-induced activation of mTOR (and presumably PI3K) in males was more transient in our studies that were conducted during the circadian light phase than what was reported in other studies that were conducted during the circadian dark phase. Regulation of circadian clock genes has been shown to influence ethanol and drug sensitivity, and ethanol also can disrupt circadian gene expression (reviewed in Parekh et al., 2015). In the present study, we did observe changes in expression of some circadian genes following binge drinking, with a significant decrease in *Per2* in females (*p* < 0.05, **Supplementary Table S1**) and trends for an opposite effect on *Clock* in females and males (females: ↑ 1.5-fold, *p* < 0.10; males: ↓ 1.5-fold, *p* < 0.09). Regardless, the results add to evidence for sex differences in the effects of binge drinking on glutamatergic signaling.

It is interesting that Akt (many times associated with PI3K) was identified as a central node in female mice following binge drinking. However, as mentioned above, we recently found that females were insensitive to the ability of intra-NAc rapamycin (inhibits mTOR, in signaling cascade downstream of PI3K and Akt) to decrease binge drinking, whereas intra-NAc rapamycin significantly decreased binge drinking in males (Cozzoli et al., 2016). The reduction in binge drinking in males is consistent with prior work (Neasta et al., 2010, 2011, 2014), so the insensitivity of females suggests that an alternate signaling pathway that is independent of PI3K and that links Group 1 mGluRs to transcriptional changes in the nucleus (i.e., protein kinase A, calcium calmodulin dependent protein kinase, or MAPK; see Wang and Zhuo, 2012) is influenced by binge drinking in females. One possible mechanism would be via the ability of membrane estrogen receptors (mER) to stimulate mGluRs, as coupling of mERα to mGluRs can initiate independent signal transduction pathways (see review by Meitzen and Mermelstein, 2011). Related to this point, the present study found that binge drinking produced a fivefold upregulation in *Esr1* (which encodes ERα) only in female mice (*p* < 0.05, **Table 3**), and evidence indicates that ERα also can localize to the plasma membrane and initiate signal transduction through PI3K (reviewed in Levin, 2009). So, it is not known whether the identification of Akt (and presumably the Akt-PI3K pathway) as a central node in females following binge drinking is related to signaling downstream of mERα or downstream from the coupling of mERα to mGluRs.

Characterization of the expression differences following binge drinking also identified hormone signaling in female mice, with a trend for regulation of CRH signaling in male mice. In fact, “CRH signaling” was identified as a canonical pathway of interest from the analysis of genes that were regulated by binge drinking in both males and females (**Figure 3**). Importantly, the effects of binge drinking on CRH signaling were divergent for all the genes listed in **Figure 3D**. An examination of the genes responsible for the initiation of CRH signaling indicate that binge drinking reduces activity of the pathway in females (↓ *Crhr1* and *Crhr2*), while it increased activity of the pathway in males (↑ *Crh* and *Crhr2*). The decreased activity of the CRH pathway in females is interesting, given evidence for sex differences in the coupling of CRHR1 with the Gs and β-arrestin 2 proteins that render females more responsive to acute stress and less able to adapt to chronic stress as a result of compromised CRHR1 internalization (Valentino et al., 2013a,b). Consistent with this, we found that 1 month of continuous ethanol drinking with intermittent traumatic stress exposure upregulated protein levels of CRHR1 in the hippocampus and protein levels of GR in the hippocampus and PFC of female but not male C57BL/6J mice (Finn et al., 2018). Taken in conjunction with the present results, it is possible that binge drinking alone produces an opposite effect on CRH signaling than the combination of stress and ethanol consumption.

The transcriptional response to repeated binge drinking identified “MIF-mediated glucocorticoid regulation” in the NAc of females in the present study, despite the divergent and non-significant binge drinking-related changes in expression of the gene encoding GR (*Nr3c1*; females: ↓ 1.1-fold, *p* = 0.19; males: ↑ 1.2-fold, *p* < 0.19) and the gene encoding the chaperone heat shock protein 90 (*Hsp90b1*; females: ↑ 2-fold, *p* < 0.08; males: ↓ 1.3-fold, *p* = 0.18). Similarly, the identification of GR signaling in the NAc and central nucleus of the amygdala of male rats (McBride et al., 2010) and an enhanced response to glucocorticoids in the VTA of female rats (McBride et al., 2013) following chronic binge drinking experience (8 weeks for male rats; 10 weeks for female rats) was based on significant binge ethanol-induced regulation of the expression of genes that did not include GR. It is well documented that glucocorticoids can act via a nuclear GR to regulate many transcriptional pathways, including homeostasis, metabolism, and inflammation (reviewed in Biddie et al., 2012). But, while glucocorticoids can have anti-inflammatory and immunosuppressive properties, long term and/or high dose glucocorticoid administration can lead to symptoms of depression and decreased immunological function. Relevant to the pathway identified in females in the present study, MIF is able to directly regulate the immunosuppressive action of glucocorticoids (reviewed in Flaster et al., 2007). MIF can be produced at all levels of the hypothalamic-pituitary-adrenal axis, and plasma MIF levels fluctuate in a circadian rhythm relative to cortisol. Early studies found that MIF counteracted the glucocorticoid-induced suppression of inflammatory cytokine secretion in activated macrophages (e.g., TNF, IL-1, IL-6, IL-8) and completely blocked the protective effect of the synthetic glucocorticoid dexamethasone in a model of lethal, endotoxic shock induced by lipopolysaccharide (Calandra et al., 1995), providing evidence that the regulatory effect of MIF on glucocorticoid immunosuppression occurs *in vivo*. Additionally, cross-talk between GR and NF-κB occurs via a physical interaction that produces a dose-dependent and mutual antagonism effect mediated by the p65 (RelA, encoded by *Rela*) subunit of NF-κB (McKay and Cidlowski, 1998). Glucocorticoids also inhibit NF-κB activation, in part by increasing the expression of the IκB complex that maintains NF-κB in an inactive state until IκB dissociates from NF-κB following its phosphorylation (Flaster et al., 2007; Lang et al., 2015; simplified NF-κB and IκB interaction depicted in **Figure 5A**). And, one effect of MIF is to prevent glucocorticoids from increasing the expression of IκB, which would offset the glucocorticoid-mediated inhibition of NF-κB (Flaster et al., 2007; Lang et al., 2015). Since the immunosuppressive and anti-inflammatory effects of glucocorticoids are thought to depend on the inhibition of NF-κB, which is a transcription factor that plays a role in immune signaling and cell survival (Li and Verma, 2002), counteracting this effect with MIF could result in sustained inflammatory signaling in females with binge drinking experience.

Acute withdrawal following chronic intoxication affected pathways related to inflammatory activation and apoptotic/cell death signaling in PFC from females vs. males (Hashimoto and Wiren, 2008; Wilhelm et al., 2014, 2015). Acute withdrawal from repeated binge drinking also affected several pathways related to immune function only in female NAc in the present study (“MIF regulation of innate immunity,” “TNFR1 signaling,” and “TNFR2 signaling”). And, “TNFR2 signaling” was identified as a canonical pathway of interest from the analysis of genes that were regulated by binge drinking in the NAc from both males and females (**Figure 5**). Notably, binge drinking produced a significant and opposite change in the expression of *Lta* (encodes TNF-β or lymphotoxin-α), with a decrease in females and increase in males (**Table 2** and **Figure 5G**), and this protein also is involved in cell survival, proliferation, differentiation, apoptosis, and immune regulation. So, the sex difference in significant expression change in *Lta* by binge drinking (↓ in females, ↑ in males, **Table 2** and **Figure 5G**) would be predicted to have opposite effects on cell survival and immune responses. In addition, binge drinking significantly decreased expression of *Il6* only in females; an ethanol-induced reduction in levels of these two cytokines in females would be consistent with a decrease in the initiation of signaling at TNFR1 (see Figure 2 in Mayfield et al., 2013) and TNFR2 (**Figure 5A**). Overall, the results in females demonstrate that binge drinking produced a fairly consistent decrease in expression of genes in the signaling cascade through the TNF superfamily, with the exception of a trend for an increase in expression of *Tnf* (encodes TNF-α). These binge ethanol-induced changes in females, including the downregulation of the RelA subunit of NF-κB, likely produce a decrease in the activation of NF-κB. A more complex pattern of changes was identified in males following binge drinking, so the influence on NF-κB activation in males is unclear. Regardless, the results in females in the present study would be consistent with a decrease in cell survival, and an increase in apoptosis and inflammation via a decrease in the activation of the NF-κB. It is interesting that acute withdrawal from chronic intoxication also identified NF-κB as a central node in both male and female networks, but the interacting gene sets were completely distinct between the sexes (Wilhelm et al., 2014). An examination of the genes regulated by chronic intoxication in these pathways revealed that several of the genes in females were indicative of a proinflammatory response, while the genes in males were suggestive of overall immunosuppression (Wilhelm et al., 2014). Collectively, binge drinking experience and chronic intoxication leading to the development of physical dependence both produced sexually divergent changes in inflammatory signaling in the NAc and PFC from mice.

## Conclusion

The repeated binge drinking sessions produced sexually divergent transcriptional responses and activation of distinct networks. These results add to a body of evidence indicating that binge drinking and chronic ethanol intoxication both produce neuroadaptive changes in neurotransmitter systems and in many cellular pathways that likely alter neuronal function in a manner that can be either adaptive or deleterious. The opposite effects of binge drinking on immune function in the present study, with changes in females consistent with a decrease in cell survival and an increase in inflammation and apoptosis, have important implications, given the evidence for a role of neuroimmune signaling in the acute and chronic effects of ethanol, including neurodegeneration (reviewed in Mayfield et al., 2013; Crews et al., 2015). Related to this point, chronic intoxication activated inflammatory signaling and cell death pathways in female but not male mice, and confirmation studies showed that ethanol dependent females exhibited significant neuronal degeneration in cortical regions, whereas cell death in males was significantly reduced (Hashimoto and Wiren, 2008; Wilhelm et al., 2015). Finally, a broader implication of the current findings is pertinent to sex differences in the immune system and the relationship to mood disorders (reviewed in Rainville and Hodes, 2018). Taken in conjunction with sex differences in mood and anxiety disorders (e.g., Altemus et al., 2014), future studies examining potential immune or stress-related mechanisms that may contribute to stress and ethanol susceptibility and associated mood disorders will be important.

One limitation of the present investigation is that we did not conduct confirmation studies to identify protein changes, neuronal degeneration, or behavioral changes that could possibly account for the sex-specific gene expression profiles that we observed. However, we did examine the expression of additional select genes that were not present on the arrays but that were implicated in the downstream signaling cascades of the IPA-identified pathways to strengthen conclusions about the select pathways that were altered by binge drinking. Future studies will determine whether the current gene expression changes correspond to behavioral and/or physiological differences.

Importantly, an increased understanding of sexually dimorphic molecular pathways influenced by binge drinking and chronic intoxication leading to dependence may identify novel treatment options for males and females. The current study is contributing data sets that can be used to generate sex-specific bioinformatics tools, which have the potential to enormously accelerate the discovery of sex-specific changes associated with AUD. Finally, we recently reported that binge drinking produced sex differences in the regulation of PI3K signaling in the NAc and in the ability of intra-NAc rapamycin to decrease binge drinking, with females resistant to these molecular changes (Cozzoli et al., 2016). The functional implication of the report by Cozzoli et al. (2016) emphasizes that targeting a pathway that is unaffected by binge drinking in females will not be an effective pharmacotherapeutic strategy. Collectively, the fundamental sex differences identified in the present and prior work provide evidence for distinct pathways that could be targeted therapeutically for the treatment of AUD in males and females.

## Data Availability

The raw data supporting the conclusions of this manuscript will be made available by the authors, without undue reservation, to any qualified researcher.

## Author Contributions

DF and KW contributed conception and study design. DF, DC, MN, and MK conducted the drinking study. DC dissected accumbens tissue. JH isolated RNA and prepared samples for qPCR array analysis. DF conducted preliminary analysis of drinking data and identified animals for the qPCR analysis. DF, MN, and MK participated in final analysis of drinking data. MH conducted follow-up qRT-PCR analysis, with assistance from JH. JH and KW performed Pathway Analysis. JH, KW, and MG assisted in the interpretation of the array and pathway analyses. DF wrote the first draft of the manuscript, but JH and KW wrote sections of the “Materials and Methods and Results.” All authors contributed to the final version of the manuscript, read, and approved the final version.

## Conflict of Interest Statement

The authors declare that the research was conducted in the absence of any commercial or financial relationships that could be construed as a potential conflict of interest.

## Footnotes

1 http://github.com/jdstorey/qvalue

2 www.ingenuity.com
