# Supplementary material for: Binge Ethanol Drinking Produces Sexually Divergent and Distinct Changes in Nucleus Accumbens Signaling Cascades and Pathways in Adult C57BL/6J Mice
Source: Front Genet. 2018 Sep 10;9:325. doi: 10.3389/fgene.2018.00325 (PMC6139464; doi:10.3389/fgene.2018.00325)
Supplement: Supplementary file 1 [file Table_1.docx]

**Supplemental Table 1. Nucleus accumbens genes significantly regulated by binge drinking in female mice.** A total of 70 genes in the nucleus accumbens were significantly regulated by 7 episodes of binge ethanol drinking in females. Regulation by binge ethanol drinking in males is shown for comparative purposes. Significance is based on *p*-values, but *q*-values also are shown. Transcripts are listed in ascending order for the female mice. Fold change of binge vs control is shown, with negative values indicating down-regulation by binge ethanol drinking and positive values indicating up-regulation by ethanol. We note that some of the genes with high fold changes (*Casp8*, *Smc4*, *Alox12*, *Pmch*, *Dgka*, *Prkcq*, *Timeless*, *Fosl1*, and *Slc6a2*) had 2 or more samples with undetected expression, indicating qualitative regulation (i.e., present in binge ethanol samples but absent in controls).

| **Gene Symbol** | **Gene Name** | **Female**  **Fold**  **change *p* value *q* value** | | | **Male**  **Fold**  **change *p* value *q* value** | | |
| --- | --- | --- | --- | --- | --- | --- | --- |
| *Casp8* | caspase 8 | 114.71 | 0.001 | 0.021 | 1.49 | 0.201 | 0.066 |
| *Smc4* | structural maintenance of chromosomes 4 | 93.99 | 0.000 | 0.018 | -2.48 | 0.017 | 0.064 |
| *Alox12* | arachidonate 12-lipoxygenase | 73.14 | 0.000 | 0.018 | 2.13 | 1 | 0.201 |
| *Pmch* | pro-melanin-concentrating hormone | 28.46 | 0.001 | 0.022 | -2.06 | 0.139 | 0.066 |
| *Dgka* | diacylglycerol kinase, alpha | 11.02 | 0.032 | 0.070 | -1.84 | 0.034 | 0.065 |
| *Prkcq* | protein kinase C, theta | 9.22 | 0.035 | 0.070 | -1.13 | 0.536 | 0.113 |
| *Timeless* | timeless circadian clock 1 | 5.33 | 0.048 | 0.081 | 1.40 | 0.406 | 0.090 |
| *Esr1* | estrogen receptor 1 (alpha) | 5.00 | 0.019 | 0.064 | 5.94 | 0.142 | 0.066 |
| *Dlx1* | distal-less homeobox 1 | 4.41 | 0.002 | 0.039 | 2.10 | 0.085 | 0.066 |
| *Eif2s2* | eukaryotic translation initiation factor 2, subunit 2 (beta) | 4.07 | 0.013 | 0.064 | -3.20 | 0.134 | 0.066 |
| *Hdac1* | histone deacetylase 1 | 3.63 | 0.012 | 0.064 | 1.03 | 0.164 | 0.066 |
| *Egfr* | epidermal growth factor receptor | 3.50 | 0.013 | 0.064 | -1.02 | 0.434 | 0.094 |
| *Pafah1b1* | platelet-activating factor acetylhydrolase, isoform 1b, subunit 1 | 2.99 | 0.015 | 0.064 | 1.08 | 0.145 | 0.066 |
| *Katnal1* | katanin p60 subunit A-like 1 | 2.98 | 0.023 | 0.070 | 1.00 | 0.248 | 0.066 |
| *Nos1* | nitric oxide synthase 1, neuronal | 2.78 | 0.042 | 0.075 | -1.91 | 0.023 | 0.064 |
| *Enpp2* | ectonucleotide pyrophosphatase/phosphodiesterase 2 | 2.76 | 0.024 | 0.070 | -1.09 | 0.194 | 0.066 |
| *Gfap* | glial fibrillary acidic protein | 2.65 | 0.037 | 0.070 | -1.43 | 0.133 | 0.066 |
| *Lpar1* | lysophosphatidic acid receptor 1 | 2.61 | 0.009 | 0.064 | -1.19 | 0.210 | 0.066 |
| *Mgat3* | mannoside acetylglucosaminyltransferase 3 | 2.58 | 0.032 | 0.070 | -1.14 | 0.192 | 0.066 |
| *Ywhah* | tyrosine 3-monooxygenase/tryptophan 5-monooxygenase activation protein, eta polypeptide | 2.43 | 0.035 | 0.070 | 1.18 | 0.233 | 0.066 |
| *Gabra3* | gamma-aminobutyric acid (GABA) A receptor, subunit alpha 3 | 2.40 | 0.035 | 0.070 | 1.39 | 0.051 | 0.066 |
| *Impa1* | inositol (myo)-1(or 4)-monophosphatase 1 | 2.33 | 0.031 | 0.070 | 1.04 | 0.238 | 0.066 |
| *Ranbp9* | RAN binding protein 9 | 2.30 | 0.036 | 0.070 | 1.59 | 0.047 | 0.066 |
| *Tcf4* | transcription factor 4 | 2.27 | 0.032 | 0.070 | 1.09 | 0.211 | 0.066 |
| *Pdlim5* | PDZ and LIM domain 5 | 2.20 | 0.044 | 0.076 | -1.20 | 0.518 | 0.109 |
| *Olig2* | oligodendrocyte transcription factor 2 | 2.14 | 0.043 | 0.075 | -1.50 | 0.091 | 0.066 |
| *Star* | steroidogenic acute regulatory protein | 1.90 | 0.042 | 0.075 | -2.18 | 0.018 | 0.064 |
| *Golga2* | golgi autoantigen, golgin subfamily a, 2 | 1.89 | 0.049 | 0.082 | -1.19 | 0.226 | 0.066 |
| *Nfkb1* | nuclear factor of kappa light polypeptide gene enhancer in B cells 1, p105 | -1.66 | 0.036 | 0.070 | -1.06 | 0.197 | 0.066 |
| *Rela* | v-rel reticuloendotheliosis viral oncogene homolog A (avian) | -1.70 | 0.036 | 0.070 | -1.04 | 0.306 | 0.074 |
| *Per2* | period circadian clock 2 | -1.75 | 0.030 | 0.070 | -1.20 | 0.175 | 0.066 |
| *Tph2* | tryptophan hydroxylase 2 | -1.79 | 0.033 | 0.070 | 1.15 | 0.402 | 0.089 |
| *Reln* | reelin | -1.95 | 0.017 | 0.064 | -2.17 | 0.011 | 0.064 |
| *Slc18a2* | solute carrier family 18 (vesicular monoamine), member 2 | -1.95 | 0.018 | 0.064 | 1.24 | 0.165 | 0.066 |
| *Drd5* | dopamine receptor D5 | -2.07 | 0.034 | 0.070 | -2.30 | 0.021 | 0.064 |
| *Crtc1* | CREB regulated transcription coactivator 1 | -2.11 | 0.027 | 0.070 | -1.38 | 0.151 | 0.066 |
| *Ckm* | creatine kinase, muscle | -2.12 | 0.015 | 0.064 | -1.02 | 0.290 | 0.072 |
| *Ndufv2* | NADH dehydrogenase (ubiquinone) flavoprotein 2 | -2.17 | 0.018 | 0.064 | -1.46 | 0.304 | 0.074 |
| *Tnnt1* | troponin T1, skeletal, slow | -2.17 | 0.028 | 0.070 | 1.02 | 0.253 | 0.066 |
| *Grm4* | glutamate receptor, metabotropic 4 | -2.22 | 0.030 | 0.070 | -2.19 | 0.019 | 0.064 |
| *Casp6* | caspase 6 | -2.38 | 0.016 | 0.064 | -1.53 | 0.247 | 0.066 |
| *Tph1* | tryptophan hydroxylase 1 | -2.42 | 0.014 | 0.064 | 1.80 | 0.420 | 0.091 |
| *Gzmb* | granzyme B | -2.46 | 0.016 | 0.064 | 1.15 | 0.181 | 0.066 |
| *Ccl2* | chemokine (C-C motif) ligand 2 | -2.48 | 0.035 | 0.070 | 1.09 | 0.270 | 0.068 |
| *Cox6c* | cytochrome c oxidase subunit VIc | -2.56 | 0.043 | 0.075 | 1.24 | 0.138 | 0.066 |
| *Clcf1* | cardiotrophin-like cytokine factor 1 | -2.65 | 0.018 | 0.064 | 1.03 | 0.343 | 0.079 |
| *Pla2g5* | phospholipase A2, group V | -2.65 | 0.019 | 0.064 | 2.24 | 0.072 | 0.066 |
| *Syn3* | synapsin III | -2.68 | 0.025 | 0.070 | -1.33 | 0.213 | 0.066 |
| *Lef1* | lymphoid enhancer binding factor 1 | -2.91 | 0.016 | 0.064 | 1.22 | 0.137 | 0.066 |
| *Crhr1* | corticotropin releasing hormone receptor 1 | -3.02 | 0.004 | 0.057 | -1.07 | 0.190 | 0.066 |
| *Pla2g3* | phospholipase A2, group III | -3.09 | 0.021 | 0.066 | -1.30 | 0.163 | 0.066 |
| *Crhr2* | corticotropin releasing hormone receptor 2 | -3.13 | 0.020 | 0.066 | 2.07 | 0.022 | 0.064 |
| *Pla2g4c* | phospholipase A2, group IVC (cytosolic, calcium-independent) | -3.19 | 0.025 | 0.070 | -1.06 | 0.259 | 0.067 |
| *Pax5* | paired box gene 5 | -3.22 | 0.018 | 0.064 | -1.55 | 0.350 | 0.079 |
| *Il6* | interleukin 6 | -3.41 | 0.012 | 0.064 | -1.28 | 0.326 | 0.077 |
| *Fos* | FBJ osteosarcoma oncogene | -3.44 | 0.004 | 0.054 | 1.64 | 0.030 | 0.065 |
| *Impa2* | inositol (myo)-1(or 4)-monophosphatase 2 | -3.53 | 0.035 | 0.070 | 1.32 | 0.140 | 0.066 |
| *Drd3* | dopamine receptor D3 | -3.53 | 0.028 | 0.070 | -2.25 | 0.069 | 0.066 |
| *Mc5r* | melanocortin 5 receptor | -3.64 | 0.003 | 0.051 | 1.93 | 0.024 | 0.064 |
| *Nos1ap* | nitric oxide synthase 1 (neuronal) adaptor protein | -3.91 | 0.010 | 0.064 | 1.43 | 0.043 | 0.066 |
| *St8sia2* | ST8 alpha-N-acetyl-neuraminide alpha-2,8-sialyltransferase 2 | -4.08 | 0.011 | 0.064 | -1.80 | 0.094 | 0.066 |
| *Nfkbib* | nuclear factor of kappa light polypeptide gene enhancer in B-cells inhibitor, beta | -4.80 | 0.012 | 0.064 | 2.57 | 0.096 | 0.066 |
| *Ppp1r1b* | protein phosphatase 1, regulatory (inhibitor) subunit 1B | -4.89 | 0.037 | 0.070 | -2.74 | 0.056 | 0.066 |
| *Il2rb* | interleukin 2 receptor, beta chain | -5.14 | 0.012 | 0.064 | -1.23 | 0.332 | 0.078 |
| *Il9r* | interleukin 9 receptor | -6.18 | 0.007 | 0.064 | 1.19 | 0.250 | 0.066 |
| *Drd4* | dopamine receptor D4 | -9.72 | 0.006 | 0.064 | 1.16 | 0.699 | 0.144 |
| *Prf1* | perforin 1 (pore forming protein) | -11.61 | 0.009 | 0.064 | -1.52 | 0.213 | 0.066 |
| *Lta* | lymphotoxin A | -14.33 | 0.001 | 0.022 | 1.88 | 0.027 | 0.064 |
| *Fosl1* | fos-like antigen 1 | -15.10 | 0.039 | 0.073 | 1.10 | 0.342 | 0.079 |
| *Slc6a2* | solute carrier family 6 (neurotransmitter transporter, noradrenalin), member 2 | -21.58 | 0.019 | 0.064 | 10.49 | 0.000 | 0.016 |
