# Supplementary material for: Binge Ethanol Drinking Produces Sexually Divergent and Distinct Changes in Nucleus Accumbens Signaling Cascades and Pathways in Adult C57BL/6J Mice
Source: Front Genet. 2018 Sep 10;9:325. doi: 10.3389/fgene.2018.00325 (PMC6139464; doi:10.3389/fgene.2018.00325)
Supplement: Supplementary file 2 [file Table_2.docx]

**Supplemental Table 2. Nucleus accumbens genes significantly regulated by binge drinking in male mice.** A total of 50 genes in the nucleus accumbens were significantly regulated by 7 binge ethanol drinking sessions in males. Regulation by binge drinking in females is shown for comparative purposes. Significance is based on *p*-values, but *q*-values also are shown. Transcripts are listed in ascending order for the male mice. Fold change of binge vs control is shown, with negative values indicating down-regulation by binge ethanol drinking and positive values indicating up-regulation by ethanol. We note that some of the genes with high fold changes (*Fasl*, *Chrna1*, *Slc6a2*, and *S100a9*) had 2 or more samples with undetected expression, indicating qualitative regulation (i.e., present in binge ethanol samples but absent in controls).

| **Gene Symbol** | **Gene Name** | **Male**  **Fold**  **change *p* value *q* value** | | | **Female**  **Fold**  **change *p* value *q* value** | | |
| --- | --- | --- | --- | --- | --- | --- | --- |
| *Fasl* | Fas ligand (TNF superfamily, member 6) | 20.15 | 0.016 | 0.064 | -3.63 | 0.111 | 0.085 |
| *Chrna1* | cholinergic receptor, nicotinic, alpha polypeptide 1 (muscle) | 15.31 | 0.019 | 0.064 | -1.20 | 0.490 | 0.157 |
| *Slc6a2* | solute carrier family 6 (neurotransmitter transporter, noradrenalin), member 2 | 10.49 | 0.000 | 0.016 | -21.58 | 0.019 | 0.064 |
| *Crh* | corticotropin releasing hormone | 5.32 | 0.041 | 0.066 | 1.71 | 0.295 | 0.103 |
| *Ptgs2* | prostaglandin-endoperoxide synthase 2 | 4.66 | 0.009 | 0.064 | -1.06 | 0.214 | 0.088 |
| *Bdnf* | brain derived neurotrophic factor | 4.26 | 0.008 | 0.064 | 1.92 | 0.193 | 0.085 |
| *Crhr2* | corticotropin releasing hormone receptor 2 | 2.07 | 0.022 | 0.064 | -3.13 | 0.020 | 0.066 |
| *Tuba8* | tubulin, alpha 8 | 1.96 | 0.016 | 0.064 | 1.07 | 0.327 | 0.112 |
| *Mc5r* | melanocortin 5 receptor | 1.93 | 0.024 | 0.064 | -3.64 | 0.003 | 0.051 |
| *Cpt2* | carnitine palmitoyltransferase 2 | 1.91 | 0.025 | 0.064 | -1.79 | 0.086 | 0.085 |
| *Lta* | lymphotoxin A | 1.88 | 0.027 | 0.064 | -14.33 | 0.001 | 0.022 |
| *Dusp6* | dual specificity phosphatase 6 | 1.86 | 0.023 | 0.064 | 1.09 | 0.260 | 0.096 |
| *Homer2* | homer homolog 2 (Drosophila) | 1.83 | 0.027 | 0.064 | -2.74 | 0.425 | 0.138 |
| *Slc6a3* | solute carrier family 6 (neurotransmitter transporter, dopamine), member 3 | 1.80 | 0.028 | 0.064 | 1.13 | 0.477 | 0.153 |
| *Gabra1* | gamma-aminobutyric acid (GABA) A receptor, subunit alpha 1 | 1.67 | 0.045 | 0.066 | 1.65 | 0.163 | 0.085 |
| *Fos* | FBJ osteosarcoma oncogene | 1.64 | 0.030 | 0.065 | -3.44 | 0.004 | 0.054 |
| *Ranbp9* | RAN binding protein 9 | 1.59 | 0.047 | 0.066 | 2.30 | 0.036 | 0.070 |
| *Maoa* | monoamine oxidase A | 1.58 | 0.045 | 0.066 | -1.05 | 0.201 | 0.085 |
| *Ccnd1* | cyclin D1 | 1.58 | 0.044 | 0.066 | 2.59 | 0.096 | 0.085 |
| *Nos1ap* | nitric oxide synthase 1 (neuronal) adaptor protein | 1.43 | 0.043 | 0.066 | -3.91 | 0.010 | 0.064 |
| *Kcnq2* | potassium voltage-gated channel, subfamily Q, member 2 | -1.66 | 0.046 | 0.066 | -1.03 | 0.182 | 0.085 |
| *Nedd4l* | neural precursor cell expressed, developmentally down-regulated gene 4-like | -1.75 | 0.033 | 0.065 | 1.08 | 0.219 | 0.088 |
| *G6pdx* | glucose-6-phosphate dehydrogenase X-linked | -1.75 | 0.047 | 0.066 | -1.48 | 0.093 | 0.085 |
| *Mthfd1* | methylenetetrahydrofolate dehydrogenase (NADP+ dependent), methenyltetrahydrofolate cyclohydrolase, formyltetrahydrofolate synthase | -1.80 | 0.028 | 0.064 | 1.21 | 0.178 | 0.085 |
| *Gria1* | glutamate receptor, ionotropic, AMPA1 (alpha 1) | -1.84 | 0.027 | 0.064 | 1.62 | 0.088 | 0.085 |
| *Dgka* | diacylglycerol kinase, alpha | -1.84 | 0.034 | 0.065 | 11.02 | 0.032 | 0.070 |
| *Mthfr* | 5,10-methylenetetrahydrofolate reductase | -1.87 | 0.027 | 0.064 | 1.91 | 0.125 | 0.085 |
| *Gpx2* | glutathione peroxidase 2 | -1.88 | 0.041 | 0.066 | -3.18 | 0.245 | 0.093 |
| *Nos1* | nitric oxide synthase 1, neuronal | -1.91 | 0.023 | 0.064 | 2.78 | 0.042 | 0.075 |
| *Scg2* | secretogranin II | -1.92 | 0.022 | 0.064 | 1.18 | 0.218 | 0.088 |
| *Pde4b* | phosphodiesterase 4B, cAMP specific | -1.96 | 0.017 | 0.064 | 1.36 | 0.351 | 0.118 |
| *Trpc7* | transient receptor potential cation channel, subfamily C, member 7 | -1.99 | 0.017 | 0.064 | -4.56 | 0.121 | 0.085 |
| *Actn2* | actinin alpha 2 | -2.10 | 0.025 | 0.064 | -1.12 | 0.164 | 0.085 |
| *Reln* | reelin | -2.17 | 0.011 | 0.064 | -1.95 | 0.017 | 0.064 |
| *Star* | steroidogenic acute regulatory protein | -2.18 | 0.018 | 0.064 | 1.90 | 0.042 | 0.075 |
| *Grm4* | glutamate receptor, metabotropic 4 | -2.19 | 0.019 | 0.064 | -2.22 | 0.030 | 0.070 |
| *Npas2* | neuronal PAS domain protein 2 | -2.20 | 0.011 | 0.064 | -1.47 | 0.097 | 0.085 |
| *Drd5* | dopamine receptor D5 | -2.30 | 0.021 | 0.064 | -2.07 | 0.034 | 0.070 |
| *Rap1gap* | Rap1 GTPase-activating protein | -2.37 | 0.033 | 0.065 | -1.19 | 0.232 | 0.091 |
| *Ccl3* | chemokine (C-C motif) ligand 3 | -2.40 | 0.049 | 0.066 | -1.06 | 1 | 0.306 |
| *Smc4* | structural maintenance of chromosomes 4 | -2.48 | 0.017 | 0.064 | 93.99 | 0.000 | 0.018 |
| *Bcr* | breakpoint cluster region | -2.52 | 0.030 | 0.065 | -1.21 | 0.123 | 0.085 |
| *Htr1b* | 5-hydroxytryptamine (serotonin) receptor 1B | -2.80 | 0.022 | 0.064 | -2.97 | 0.064 | 0.085 |
| *Isyna1* | myo-inositol 1-phosphate synthase A1 | -2.88 | 0.015 | 0.064 | -3.04 | 0.150 | 0.085 |
| *Ace* | angiotensin I converting enzyme (peptidyl-dipeptidase A) 1 | -3.19 | 0.023 | 0.064 | 2.95 | 0.128 | 0.085 |
| *Bax* | BCL2-associated X protein | -3.47 | 0.032 | 0.065 | 1.45 | 0.098 | 0.085 |
| *Drd1* | Dopamine receptor D1 | -3.51 | 0.011 | 0.064 | -1.35 | 0.200 | 0.085 |
| *Cit* | citron | -3.96 | 0.010 | 0.064 | 2.95 | 0.353 | 0.118 |
| *Tfap2b* | transcription factor AP-2 beta | -15.01 | 0.000 | 0.013 | -2.82 | 0.059 | 0.085 |
| *S100a9* | S100 calcium binding protein A9 (calgranulin B) | -24.14 | 0.026 | 0.064 | -5.35 | 0.120 | 0.085 |
