# Supplementary material for: Binge Ethanol Drinking Produces Sexually Divergent and Distinct Changes in Nucleus Accumbens Signaling Cascades and Pathways in Adult C57BL/6J Mice
Source: Front Genet. 2018 Sep 10;9:325. doi: 10.3389/fgene.2018.00325 (PMC6139464; doi:10.3389/fgene.2018.00325)
Supplement: Supplementary file 3 [file Table_3.DOCX]

**Supplemental Table 3. Customized Mood Disorder Array Genes.** A total of 384 genes were on the Mood Disorder Array. Gene symbols and names reflect 2018 versions. *Gene ID 14968 replaced with Gene ID 100504404; gene name and symbol were updated.

| **Gene ID** | **Gene Symbol** | **Gene Name** |
| --- | --- | --- |
| 19791 | [*Rn18s*](http://www.ncbi.nlm.nih.gov/sites/entrez?Db=gene&Cmd=ShowDetailView&TermToSearch=19791) | 18S ribosomal RNA |
| 12799 | [*Cnp*](http://www.ncbi.nlm.nih.gov/sites/entrez?Db=gene&Cmd=ShowDetailView&TermToSearch=12799) | 2',3'-cyclic nucleotide 3' phosphodiesterase |
| 15562 | [*Htr4*](http://www.ncbi.nlm.nih.gov/sites/entrez?Db=gene&Cmd=ShowDetailView&TermToSearch=15562) | 5 hydroxytryptamine (serotonin) receptor 4 |
| 17769 | [*Mthfr*](http://www.ncbi.nlm.nih.gov/sites/entrez?Db=gene&Cmd=ShowDetailView&TermToSearch=17769) | 5,10-methylenetetrahydrofolate reductase |
| 15550 | [*Htr1a*](http://www.ncbi.nlm.nih.gov/sites/entrez?Db=gene&Cmd=ShowDetailView&TermToSearch=15550) | 5-hydroxytryptamine (serotonin) receptor 1A |
| 15551 | [*Htr1b*](http://www.ncbi.nlm.nih.gov/sites/entrez?Db=gene&Cmd=ShowDetailView&TermToSearch=15551) | 5-hydroxytryptamine (serotonin) receptor 1B |
| 15552 | [*Htr1d*](http://www.ncbi.nlm.nih.gov/sites/entrez?Db=gene&Cmd=ShowDetailView&TermToSearch=15552) | 5-hydroxytryptamine (serotonin) receptor 1D |
| 15558 | [*Htr2a*](http://www.ncbi.nlm.nih.gov/sites/entrez?Db=gene&Cmd=ShowDetailView&TermToSearch=15558) | 5-hydroxytryptamine (serotonin) receptor 2A |
| 15560 | [*Htr2c*](http://www.ncbi.nlm.nih.gov/sites/entrez?Db=gene&Cmd=ShowDetailView&TermToSearch=15560) | 5-hydroxytryptamine (serotonin) receptor 2C |
| 15561 | [*Htr3a*](http://www.ncbi.nlm.nih.gov/sites/entrez?Db=gene&Cmd=ShowDetailView&TermToSearch=15561) | 5-hydroxytryptamine (serotonin) receptor 3A |
| 238505 | [*Mtr*](http://www.ncbi.nlm.nih.gov/sites/entrez?Db=gene&Cmd=ShowDetailView&TermToSearch=238505) | 5-methyltetrahydrofolate-homocysteine methyltransferase |
| 210009 | [*Mtrr*](http://www.ncbi.nlm.nih.gov/sites/entrez?Db=gene&Cmd=ShowDetailView&TermToSearch=210009) | 5-methyltetrahydrofolate-homocysteine methyltransferase reductase |
| 11472 | [*Actn2*](http://www.ncbi.nlm.nih.gov/sites/entrez?Db=gene&Cmd=ShowDetailView&TermToSearch=11472) | actinin alpha 2 |
| 11911 | [*Atf4*](http://www.ncbi.nlm.nih.gov/sites/entrez?Db=gene&Cmd=ShowDetailView&TermToSearch=11911) | activating transcription factor 4 |
| 107503 | [*Atf5*](http://www.ncbi.nlm.nih.gov/sites/entrez?Db=gene&Cmd=ShowDetailView&TermToSearch=107503) | activating transcription factor 5 |
| 54343 | [*Atf7ip*](http://www.ncbi.nlm.nih.gov/sites/entrez?Db=gene&Cmd=ShowDetailView&TermToSearch=54343) | activating transcription factor 7 interacting protein |
| 110532 | [*Adarb1*](http://www.ncbi.nlm.nih.gov/sites/entrez?Db=gene&Cmd=ShowDetailView&TermToSearch=110532) | adenosine deaminase, RNA-specific, B1 |
| 11515 | [*Adcy9*](http://www.ncbi.nlm.nih.gov/sites/entrez?Db=gene&Cmd=ShowDetailView&TermToSearch=11515) | adenylate cyclase 9 |
| 11516 | [*Adcyap1*](http://www.ncbi.nlm.nih.gov/sites/entrez?Db=gene&Cmd=ShowDetailView&TermToSearch=11516) | adenylate cyclase activating polypeptide 1 |
| 11566 | [*Adss*](http://www.ncbi.nlm.nih.gov/sites/entrez?Db=gene&Cmd=ShowDetailView&TermToSearch=11566) | adenylosuccinate synthetase, non muscle |
| 320129 | [*Adrbk2*](http://www.ncbi.nlm.nih.gov/sites/entrez?Db=gene&Cmd=ShowDetailView&TermToSearch=320129) | adrenergic receptor kinase, beta 2 |
| 11548 | [*Adra1b*](http://www.ncbi.nlm.nih.gov/sites/entrez?Db=gene&Cmd=ShowDetailView&TermToSearch=11548) | adrenergic receptor, alpha 1b |
| 11835 | [*Ar*](http://www.ncbi.nlm.nih.gov/sites/entrez?Db=gene&Cmd=ShowDetailView&TermToSearch=11835) | androgen receptor |
| 11421 | [*Ace*](http://www.ncbi.nlm.nih.gov/sites/entrez?Db=gene&Cmd=ShowDetailView&TermToSearch=11421) | angiotensin I converting enzyme (peptidyl-dipeptidase A) 1 |
| 108857 | [*Ankhd1*](http://www.ncbi.nlm.nih.gov/sites/entrez?Db=gene&Cmd=ShowDetailView&TermToSearch=108857) | ankyrin repeat and KH domain containing 1 |
| 11816 | [*Apoe*](http://www.ncbi.nlm.nih.gov/sites/entrez?Db=gene&Cmd=ShowDetailView&TermToSearch=11816) | apolipoprotein E |
| 239552 | [*Apol8*](http://www.ncbi.nlm.nih.gov/sites/entrez?Db=gene&Cmd=ShowDetailView&TermToSearch=239552) | apolipoprotein L 8 |
| 11783 | [*Apaf1*](http://www.ncbi.nlm.nih.gov/sites/entrez?Db=gene&Cmd=ShowDetailView&TermToSearch=11783) | apoptotic peptidase activating factor 1 |
| 11684 | [*Alox12*](http://www.ncbi.nlm.nih.gov/sites/entrez?Db=gene&Cmd=ShowDetailView&TermToSearch=11684) | arachidonate 12-lipoxygenase |
| 11689 | [*Alox5*](http://www.ncbi.nlm.nih.gov/sites/entrez?Db=gene&Cmd=ShowDetailView&TermToSearch=11689) | arachidonate 5-lipoxygenase |
| 26361 | [*Avpr1b*](http://www.ncbi.nlm.nih.gov/sites/entrez?Db=gene&Cmd=ShowDetailView&TermToSearch=26361) | arginine vasopressin receptor 1B |
| 11865 | [*Arntl*](http://www.ncbi.nlm.nih.gov/sites/entrez?Db=gene&Cmd=ShowDetailView&TermToSearch=11865) | aryl hydrocarbon receptor nuclear translocator-like |
| 102580 | [*Alg9*](http://www.ncbi.nlm.nih.gov/sites/entrez?Db=gene&Cmd=ShowDetailView&TermToSearch=102580) | asparagine-linked glycosylation 9 (alpha 1,2 mannosyltransferase) |
| 11484 | [*Aspa*](http://www.ncbi.nlm.nih.gov/sites/entrez?Db=gene&Cmd=ShowDetailView&TermToSearch=11484) | aspartoacylase |
| 11920 | [*Atm*](http://www.ncbi.nlm.nih.gov/sites/entrez?Db=gene&Cmd=ShowDetailView&TermToSearch=11920) | ataxia telangiectasia mutated |
| 20238 | [*Atxn1*](http://www.ncbi.nlm.nih.gov/sites/entrez?Db=gene&Cmd=ShowDetailView&TermToSearch=20238) | ataxin 1 |
| 228033 | [*Atp5g3*](http://www.ncbi.nlm.nih.gov/sites/entrez?Db=gene&Cmd=ShowDetailView&TermToSearch=228033) | ATP synthase, H+ transporting, mitochondrial F0 complex, subunit C3 (subunit 9) |
| 11957 | [*Atp5j*](http://www.ncbi.nlm.nih.gov/sites/entrez?Db=gene&Cmd=ShowDetailView&TermToSearch=11957) | ATP synthase, H+ transporting, mitochondrial F0 complex, subunit F |
| 11949 | [*Atp5c1*](http://www.ncbi.nlm.nih.gov/sites/entrez?Db=gene&Cmd=ShowDetailView&TermToSearch=11949) | ATP synthase, H+ transporting, mitochondrial F1 complex, gamma polypeptide 1 |
| 11307 | [*Abcg1*](http://www.ncbi.nlm.nih.gov/sites/entrez?Db=gene&Cmd=ShowDetailView&TermToSearch=11307) | ATP-binding cassette, sub-family G (WHITE), member 1 |
| 11938 | [*Atp2a2*](http://www.ncbi.nlm.nih.gov/sites/entrez?Db=gene&Cmd=ShowDetailView&TermToSearch=11938) | ATPase, Ca++ transporting, cardiac muscle, slow twitch 2 |
| 232975 | [*Atp1a3*](http://www.ncbi.nlm.nih.gov/sites/entrez?Db=gene&Cmd=ShowDetailView&TermToSearch=232975) | ATPase, Na+/K+ transporting, alpha 3 polypeptide |
| 12043 | [*Bcl2*](http://www.ncbi.nlm.nih.gov/sites/entrez?Db=gene&Cmd=ShowDetailView&TermToSearch=12043) | B cell leukemia/lymphoma 2 |
| 12018 | [*Bak1*](http://www.ncbi.nlm.nih.gov/sites/entrez?Db=gene&Cmd=ShowDetailView&TermToSearch=12018) | BCL2-antagonist/killer 1 |
| 12028 | [*Bax*](http://www.ncbi.nlm.nih.gov/sites/entrez?Db=gene&Cmd=ShowDetailView&TermToSearch=12028) | BCL2-associated X protein |
| 12122 | [*Bid*](http://www.ncbi.nlm.nih.gov/sites/entrez?Db=gene&Cmd=ShowDetailView&TermToSearch=12122) | BH3 interacting domain death agonist |
| 23827 | [*Bpnt1*](http://www.ncbi.nlm.nih.gov/sites/entrez?Db=gene&Cmd=ShowDetailView&TermToSearch=23827) | bisphosphate 3'-nucleotidase 1 |
| 12151 | [*Bmi1*](http://www.ncbi.nlm.nih.gov/sites/entrez?Db=gene&Cmd=ShowDetailView&TermToSearch=12151) | Bmi1 polycomb ring finger oncogene |
| 12064 | [*Bdnf*](http://www.ncbi.nlm.nih.gov/sites/entrez?Db=gene&Cmd=ShowDetailView&TermToSearch=12064) | brain derived neurotrophic factor |
| 110279 | [*Bcr*](http://www.ncbi.nlm.nih.gov/sites/entrez?Db=gene&Cmd=ShowDetailView&TermToSearch=110279) | breakpoint cluster region |
| 17289 | [*Mertk*](http://www.ncbi.nlm.nih.gov/sites/entrez?Db=gene&Cmd=ShowDetailView&TermToSearch=17289) | c-mer proto-oncogene tyrosine kinase |
| 13016 | [*Ctbp1*](http://www.ncbi.nlm.nih.gov/sites/entrez?Db=gene&Cmd=ShowDetailView&TermToSearch=13016) | C-terminal binding protein 1 |
| 207565 | [*Camkk2*](http://www.ncbi.nlm.nih.gov/sites/entrez?Db=gene&Cmd=ShowDetailView&TermToSearch=207565) | calcium/calmodulin-dependent protein kinase kinase 2, beta |
| 12317 | [*Calr*](http://www.ncbi.nlm.nih.gov/sites/entrez?Db=gene&Cmd=ShowDetailView&TermToSearch=12317) | calreticulin |
| 12912 | [*Creb1*](http://www.ncbi.nlm.nih.gov/sites/entrez?Db=gene&Cmd=ShowDetailView&TermToSearch=12912) | cAMP responsive element binding protein 1 |
| 56708 | [*Clcf1*](http://www.ncbi.nlm.nih.gov/sites/entrez?Db=gene&Cmd=ShowDetailView&TermToSearch=56708) | cardiotrophin-like cytokine factor 1 |
| 12896 | [*Cpt2*](http://www.ncbi.nlm.nih.gov/sites/entrez?Db=gene&Cmd=ShowDetailView&TermToSearch=12896) | carnitine palmitoyltransferase 2 |
| 12366 | [*Casp2*](http://www.ncbi.nlm.nih.gov/sites/entrez?Db=gene&Cmd=ShowDetailView&TermToSearch=12366) | caspase 2 |
| 12368 | [*Casp6*](http://www.ncbi.nlm.nih.gov/sites/entrez?Db=gene&Cmd=ShowDetailView&TermToSearch=12368) | caspase 6 |
| 12370 | [*Casp8*](http://www.ncbi.nlm.nih.gov/sites/entrez?Db=gene&Cmd=ShowDetailView&TermToSearch=12370) | caspase 8 |
| 12846 | [*Comt*](http://www.ncbi.nlm.nih.gov/sites/entrez?Db=gene&Cmd=ShowDetailView&TermToSearch=12846) | catechol-O-methyltransferase |
| 216274 | [*Cep290*](http://www.ncbi.nlm.nih.gov/sites/entrez?Db=gene&Cmd=ShowDetailView&TermToSearch=216274) | centrosomal protein 290 |
| 20296 | [*Ccl2*](http://www.ncbi.nlm.nih.gov/sites/entrez?Db=gene&Cmd=ShowDetailView&TermToSearch=20296) | chemokine (C-C motif) ligand 2 |
| 20302 | [*Ccl3*](http://www.ncbi.nlm.nih.gov/sites/entrez?Db=gene&Cmd=ShowDetailView&TermToSearch=20302) | chemokine (C-C motif) ligand 3 |
| 14825 | [*Cxcl1*](http://www.ncbi.nlm.nih.gov/sites/entrez?Db=gene&Cmd=ShowDetailView&TermToSearch=14825) | chemokine (C-X-C motif) ligand 1 |
| 20312 | [*Cx3cl1*](http://www.ncbi.nlm.nih.gov/sites/entrez?Db=gene&Cmd=ShowDetailView&TermToSearch=20312) | chemokine (C-X3-C motif) ligand 1 |
| 12425 | [*Cckar*](http://www.ncbi.nlm.nih.gov/sites/entrez?Db=gene&Cmd=ShowDetailView&TermToSearch=12425) | cholecystokinin A receptor |
| 243764 | [*Chrm2*](http://www.ncbi.nlm.nih.gov/sites/entrez?Db=gene&Cmd=ShowDetailView&TermToSearch=243764) | cholinergic receptor, muscarinic 2, cardiac |
| 11435 | [*Chrna1*](http://www.ncbi.nlm.nih.gov/sites/entrez?Db=gene&Cmd=ShowDetailView&TermToSearch=11435) | cholinergic receptor, nicotinic, alpha polypeptide 1 (muscle) |
| 11441 | [*Chrna7*](http://www.ncbi.nlm.nih.gov/sites/entrez?Db=gene&Cmd=ShowDetailView&TermToSearch=11441) | cholinergic receptor, nicotinic, alpha polypeptide 7 |
| 67064 | [*Chmp1b*](http://www.ncbi.nlm.nih.gov/sites/entrez?Db=gene&Cmd=ShowDetailView&TermToSearch=67064) | charged multivesicular body protein 1B |
| 12416 | [*Cbx2*](http://www.ncbi.nlm.nih.gov/sites/entrez?Db=gene&Cmd=ShowDetailView&TermToSearch=12416) | chromobox 2 |
| 12753 | [*Clock*](http://www.ncbi.nlm.nih.gov/sites/entrez?Db=gene&Cmd=ShowDetailView&TermToSearch=12753) | circadian locomoter output cycles kaput |
| 12704 | [*Cit*](http://www.ncbi.nlm.nih.gov/sites/entrez?Db=gene&Cmd=ShowDetailView&TermToSearch=12704) | citron |
| 18417 | [*Cldn11*](http://www.ncbi.nlm.nih.gov/sites/entrez?Db=gene&Cmd=ShowDetailView&TermToSearch=18417) | claudin 11 |
| 12675 | [*Chuk*](http://www.ncbi.nlm.nih.gov/sites/entrez?Db=gene&Cmd=ShowDetailView&TermToSearch=12675) | conserved helix-loop-helix ubiquitous kinase |
| 12918 | [*Crh*](http://www.ncbi.nlm.nih.gov/sites/entrez?Db=gene&Cmd=ShowDetailView&TermToSearch=12918) | corticotropin releasing hormone |
| 12919 | [*Crhbp*](http://www.ncbi.nlm.nih.gov/sites/entrez?Db=gene&Cmd=ShowDetailView&TermToSearch=12919) | corticotropin releasing hormone binding protein |
| 12921 | [*Crhr1*](http://www.ncbi.nlm.nih.gov/sites/entrez?Db=gene&Cmd=ShowDetailView&TermToSearch=12921) | corticotropin releasing hormone receptor 1 |
| 12922 | [*Crhr2*](http://www.ncbi.nlm.nih.gov/sites/entrez?Db=gene&Cmd=ShowDetailView&TermToSearch=12922) | corticotropin releasing hormone receptor 2 |
| 12716 | [*Ckmt1*](http://www.ncbi.nlm.nih.gov/sites/entrez?Db=gene&Cmd=ShowDetailView&TermToSearch=12716) | creatine kinase, mitochondrial 1, ubiquitous |
| 12715 | [*Ckm*](http://www.ncbi.nlm.nih.gov/sites/entrez?Db=gene&Cmd=ShowDetailView&TermToSearch=12715) | creatine kinase, muscle |
| 12914 | [*Crebbp*](http://www.ncbi.nlm.nih.gov/sites/entrez?Db=gene&Cmd=ShowDetailView&TermToSearch=12914) | CREB binding protein |
| 382056 | [*Crtc1*](http://www.ncbi.nlm.nih.gov/sites/entrez?Db=gene&Cmd=ShowDetailView&TermToSearch=382056) | CREB regulated transcription coactivator 1 |
| 13048 | [*Cux2*](http://www.ncbi.nlm.nih.gov/sites/entrez?Db=gene&Cmd=ShowDetailView&TermToSearch=13048) | cut-like homeobox 2 |
| 12443 | [*Ccnd1*](http://www.ncbi.nlm.nih.gov/sites/entrez?Db=gene&Cmd=ShowDetailView&TermToSearch=12443) | cyclin D1 |
| 12444 | [*Ccnd2*](http://www.ncbi.nlm.nih.gov/sites/entrez?Db=gene&Cmd=ShowDetailView&TermToSearch=12444) | cyclin D2 |
| 12445 | [*Ccnd3*](http://www.ncbi.nlm.nih.gov/sites/entrez?Db=gene&Cmd=ShowDetailView&TermToSearch=12445) | cyclin D3 |
| 12858 | [*Cox5a*](http://www.ncbi.nlm.nih.gov/sites/entrez?Db=gene&Cmd=ShowDetailView&TermToSearch=12858) | cytochrome c oxidase subunit Va |
| 12864 | [*Cox6c*](http://www.ncbi.nlm.nih.gov/sites/entrez?Db=gene&Cmd=ShowDetailView&TermToSearch=12864) | cytochrome c oxidase subunit VIc |
| 13170 | [*Dbp*](http://www.ncbi.nlm.nih.gov/sites/entrez?Db=gene&Cmd=ShowDetailView&TermToSearch=13170) | D site albumin promoter binding protein |
| 13142 | [*Dao*](http://www.ncbi.nlm.nih.gov/sites/entrez?Db=gene&Cmd=ShowDetailView&TermToSearch=13142) | D-amino acid oxidase |
| 23856 | [*Dido1*](http://www.ncbi.nlm.nih.gov/sites/entrez?Db=gene&Cmd=ShowDetailView&TermToSearch=23856) | death inducer-obliterator 1 |
| 105445 | [*Dock9*](http://www.ncbi.nlm.nih.gov/sites/entrez?Db=gene&Cmd=ShowDetailView&TermToSearch=105445) | dedicator of cytokinesis 9 |
| 13371 | [*Dio2*](http://www.ncbi.nlm.nih.gov/sites/entrez?Db=gene&Cmd=ShowDetailView&TermToSearch=13371) | deiodinase, iodothyronine, type II |
| 13139 | [*Dgka*](http://www.ncbi.nlm.nih.gov/sites/entrez?Db=gene&Cmd=ShowDetailView&TermToSearch=13139) | diacylglycerol kinase, alpha |
| 380921 | [*Dgkh*](http://www.ncbi.nlm.nih.gov/sites/entrez?Db=gene&Cmd=ShowDetailView&TermToSearch=380921) | diacylglycerol kinase, eta |
| 12934 | [*Dpysl2*](http://www.ncbi.nlm.nih.gov/sites/entrez?Db=gene&Cmd=ShowDetailView&TermToSearch=12934) | dihydropyrimidinase-like 2 |
| 13385 | [*Dlg4*](http://www.ncbi.nlm.nih.gov/sites/entrez?Db=gene&Cmd=ShowDetailView&TermToSearch=13385) | discs, large homolog 4 (Drosophila) |
| 244667 | [*Disc1*](http://www.ncbi.nlm.nih.gov/sites/entrez?Db=gene&Cmd=ShowDetailView&TermToSearch=244667) | disrupted in schizophrenia 1 |
| 13390 | [*Dlx1*](http://www.ncbi.nlm.nih.gov/sites/entrez?Db=gene&Cmd=ShowDetailView&TermToSearch=13390) | distal-less homeobox 1 |
| 13433 | [*Dnmt1*](http://www.ncbi.nlm.nih.gov/sites/entrez?Db=gene&Cmd=ShowDetailView&TermToSearch=13433) | DNA methyltransferase (cytosine-5) 1 |
| 81489 | [*Dnajb1*](http://www.ncbi.nlm.nih.gov/sites/entrez?Db=gene&Cmd=ShowDetailView&TermToSearch=81489) | DnaJ (Hsp40) homolog, subfamily B, member 1 |
| 13489 | [*Drd2*](http://www.ncbi.nlm.nih.gov/sites/entrez?Db=gene&Cmd=ShowDetailView&TermToSearch=13489) | dopamine receptor 2 |
| 13490 | [*Drd3*](http://www.ncbi.nlm.nih.gov/sites/entrez?Db=gene&Cmd=ShowDetailView&TermToSearch=13490) | dopamine receptor 3 |
| 13491 | [*Drd4*](http://www.ncbi.nlm.nih.gov/sites/entrez?Db=gene&Cmd=ShowDetailView&TermToSearch=13491) | dopamine receptor 4 |
| 13492 | [*Drd5*](http://www.ncbi.nlm.nih.gov/sites/entrez?Db=gene&Cmd=ShowDetailView&TermToSearch=13492) | dopamine receptor 5 |
| 13488 | [*Drd1*](http://www.ncbi.nlm.nih.gov/sites/entrez?Db=gene&Cmd=ShowDetailView&TermToSearch=13488) | dopamine receptor D1 |
| 67603 | [*Dusp6*](http://www.ncbi.nlm.nih.gov/sites/entrez?Db=gene&Cmd=ShowDetailView&TermToSearch=67603) | dual specificity phosphatase 6 |
| 94245 | [*Dtnbp1*](http://www.ncbi.nlm.nih.gov/sites/entrez?Db=gene&Cmd=ShowDetailView&TermToSearch=94245) | dystrobrevin binding protein 1 |
| 328572 | [*Ep300*](http://www.ncbi.nlm.nih.gov/sites/entrez?Db=gene&Cmd=ShowDetailView&TermToSearch=328572) | E1A binding protein p300 |
| 18606 | [*Enpp2*](http://www.ncbi.nlm.nih.gov/sites/entrez?Db=gene&Cmd=ShowDetailView&TermToSearch=18606) | ectonucleotide pyrophosphatase/phosphodiesterase 2 |
| 13626 | [*Eed*](http://www.ncbi.nlm.nih.gov/sites/entrez?Db=gene&Cmd=ShowDetailView&TermToSearch=13626) | embryonic ectoderm development |
| 13797 | [*Emx2*](http://www.ncbi.nlm.nih.gov/sites/entrez?Db=gene&Cmd=ShowDetailView&TermToSearch=13797) | empty spiracles homeobox 2 |
| 14056 | [*Ezh2*](http://www.ncbi.nlm.nih.gov/sites/entrez?Db=gene&Cmd=ShowDetailView&TermToSearch=14056) | enhancer of zeste homolog 2 (Drosophila) |
| 13649 | [*Egfr*](http://www.ncbi.nlm.nih.gov/sites/entrez?Db=gene&Cmd=ShowDetailView&TermToSearch=13649) | epidermal growth factor receptor |
| 13829 | [*Dmtn*](http://www.ncbi.nlm.nih.gov/sites/entrez?Db=gene&Cmd=ShowDetailView&TermToSearch=13829) | dematin actin binding protein |
| 13982 | [*Esr1*](http://www.ncbi.nlm.nih.gov/sites/entrez?Db=gene&Cmd=ShowDetailView&TermToSearch=13982) | estrogen receptor 1 (alpha) |
| 13983 | [*Esr2*](http://www.ncbi.nlm.nih.gov/sites/entrez?Db=gene&Cmd=ShowDetailView&TermToSearch=13983) | estrogen receptor 2 (beta) |
| 13665 | [*Eif2s1*](http://www.ncbi.nlm.nih.gov/sites/entrez?Db=gene&Cmd=ShowDetailView&TermToSearch=13665) | eukaryotic translation initiation factor 2, subunit 1 alpha |
| 67204 | [*Eif2s2*](http://www.ncbi.nlm.nih.gov/sites/entrez?Db=gene&Cmd=ShowDetailView&TermToSearch=67204) | eukaryotic translation initiation factor 2, subunit 2 (beta) |
| 217715 | [*Eif2b2*](http://www.ncbi.nlm.nih.gov/sites/entrez?Db=gene&Cmd=ShowDetailView&TermToSearch=217715) | eukaryotic translation initiation factor 2B, subunit 2 beta |
| 13667 | [*Eif2b4*](http://www.ncbi.nlm.nih.gov/sites/entrez?Db=gene&Cmd=ShowDetailView&TermToSearch=13667) | eukaryotic translation initiation factor 2B, subunit 4 delta |
| 68135 | [*Eif3h*](http://www.ncbi.nlm.nih.gov/sites/entrez?Db=gene&Cmd=ShowDetailView&TermToSearch=68135) | eukaryotic translation initiation factor 3, subunit H |
| 14137 | [*Fdft1*](http://www.ncbi.nlm.nih.gov/sites/entrez?Db=gene&Cmd=ShowDetailView&TermToSearch=14137) | farnesyl diphosphate farnesyl transferase 1 |
| 14102 | [*Fas*](http://www.ncbi.nlm.nih.gov/sites/entrez?Db=gene&Cmd=ShowDetailView&TermToSearch=14102) | Fas (TNF receptor superfamily member 6) |
| 14103 | [*Fasl*](http://www.ncbi.nlm.nih.gov/sites/entrez?Db=gene&Cmd=ShowDetailView&TermToSearch=14103) | Fas ligand (TNF superfamily, member 6) |
| 235180 | [*Fez1*](http://www.ncbi.nlm.nih.gov/sites/entrez?Db=gene&Cmd=ShowDetailView&TermToSearch=235180) | fasciculation and elongation protein zeta 1 (zygin I) |
| 14107 | [*Fat1*](http://www.ncbi.nlm.nih.gov/sites/entrez?Db=gene&Cmd=ShowDetailView&TermToSearch=14107) | FAT tumor suppressor homolog 1 (Drosophila) |
| 14281 | [*Fos*](http://www.ncbi.nlm.nih.gov/sites/entrez?Db=gene&Cmd=ShowDetailView&TermToSearch=14281) | FBJ osteosarcoma oncogene |
| 14229 | [*Fkbp5*](http://www.ncbi.nlm.nih.gov/sites/entrez?Db=gene&Cmd=ShowDetailView&TermToSearch=14229) | FK506 binding protein 5 |
| 14283 | [*Fosl1*](http://www.ncbi.nlm.nih.gov/sites/entrez?Db=gene&Cmd=ShowDetailView&TermToSearch=14283) | fos-like antigen 1 |
| 14365 | [*Fzd3*](http://www.ncbi.nlm.nih.gov/sites/entrez?Db=gene&Cmd=ShowDetailView&TermToSearch=14365) | frizzled homolog 3 (Drosophila) |
| 14765 | [*Gpr50*](http://www.ncbi.nlm.nih.gov/sites/entrez?Db=gene&Cmd=ShowDetailView&TermToSearch=14765) | G-protein-coupled receptor 50 |
| 14420 | [*Galc*](http://www.ncbi.nlm.nih.gov/sites/entrez?Db=gene&Cmd=ShowDetailView&TermToSearch=14420) | galactosylceramidase |
| 14394 | [*Gabra1*](http://www.ncbi.nlm.nih.gov/sites/entrez?Db=gene&Cmd=ShowDetailView&TermToSearch=14394) | gamma-aminobutyric acid (GABA) A receptor, subunit alpha 1 |
| 14396 | [*Gabra3*](http://www.ncbi.nlm.nih.gov/sites/entrez?Db=gene&Cmd=ShowDetailView&TermToSearch=14396) | gamma-aminobutyric acid (GABA) A receptor, subunit alpha 3 |
| 110886 | [*Gabra5*](http://www.ncbi.nlm.nih.gov/sites/entrez?Db=gene&Cmd=ShowDetailView&TermToSearch=110886) | gamma-aminobutyric acid (GABA) A receptor, subunit alpha 5 |
| 14406 | [*Gabrg2*](http://www.ncbi.nlm.nih.gov/sites/entrez?Db=gene&Cmd=ShowDetailView&TermToSearch=14406) | gamma-aminobutyric acid (GABA) A receptor, subunit gamma 2 |
| -3 | [*genomic3*](http://www.ncbi.nlm.nih.gov/sites/entrez?Db=gene&Cmd=ShowDetailView&TermToSearch=-3) |  |
| 14580 | [*Gfap*](http://www.ncbi.nlm.nih.gov/sites/entrez?Db=gene&Cmd=ShowDetailView&TermToSearch=14580) | glial fibrillary acidic protein |
| 14381 | [*G6pdx*](http://www.ncbi.nlm.nih.gov/sites/entrez?Db=gene&Cmd=ShowDetailView&TermToSearch=14381) | glucose-6-phosphate dehydrogenase X-linked |
| 14799 | [*Gria1*](http://www.ncbi.nlm.nih.gov/sites/entrez?Db=gene&Cmd=ShowDetailView&TermToSearch=14799) | glutamate receptor, ionotropic, AMPA1 (alpha 1) |
| 14807 | [*Grik3*](http://www.ncbi.nlm.nih.gov/sites/entrez?Db=gene&Cmd=ShowDetailView&TermToSearch=14807) | glutamate receptor, ionotropic, kainate 3 |
| 110637 | [*Grik4*](http://www.ncbi.nlm.nih.gov/sites/entrez?Db=gene&Cmd=ShowDetailView&TermToSearch=110637) | glutamate receptor, ionotropic, kainate 4 |
| 14810 | [*Grin1*](http://www.ncbi.nlm.nih.gov/sites/entrez?Db=gene&Cmd=ShowDetailView&TermToSearch=14810) | glutamate receptor, ionotropic, NMDA1 (zeta 1) |
| 14811 | [*Grin2a*](http://www.ncbi.nlm.nih.gov/sites/entrez?Db=gene&Cmd=ShowDetailView&TermToSearch=14811) | glutamate receptor, ionotropic, NMDA2A (epsilon 1) |
| 14812 | [*Grin2b*](http://www.ncbi.nlm.nih.gov/sites/entrez?Db=gene&Cmd=ShowDetailView&TermToSearch=14812) | glutamate receptor, ionotropic, NMDA2B (epsilon 2) |
| 14814 | [*Grin2d*](http://www.ncbi.nlm.nih.gov/sites/entrez?Db=gene&Cmd=ShowDetailView&TermToSearch=14814) | glutamate receptor, ionotropic, NMDA2D (epsilon 4) |
| 108069 | [*Grm3*](http://www.ncbi.nlm.nih.gov/sites/entrez?Db=gene&Cmd=ShowDetailView&TermToSearch=108069) | glutamate receptor, metabotropic 3 |
| 268934 | [*Grm4*](http://www.ncbi.nlm.nih.gov/sites/entrez?Db=gene&Cmd=ShowDetailView&TermToSearch=268934) | glutamate receptor, metabotropic 4 |
| 108071 | [*Grm5*](http://www.ncbi.nlm.nih.gov/sites/entrez?Db=gene&Cmd=ShowDetailView&TermToSearch=108071) | glutamate receptor, metabotropic 5 |
| 14415 | [*Gad1*](http://www.ncbi.nlm.nih.gov/sites/entrez?Db=gene&Cmd=ShowDetailView&TermToSearch=14415) | glutamic acid decarboxylase 1 |
| 14775 | [*Gpx1*](http://www.ncbi.nlm.nih.gov/sites/entrez?Db=gene&Cmd=ShowDetailView&TermToSearch=14775) | glutathione peroxidase 1 |
| 14776 | [*Gpx2*](http://www.ncbi.nlm.nih.gov/sites/entrez?Db=gene&Cmd=ShowDetailView&TermToSearch=14776) | glutathione peroxidase 2 |
| 625249 | [*Gpx4*](http://www.ncbi.nlm.nih.gov/sites/entrez?Db=gene&Cmd=ShowDetailView&TermToSearch=625249) | glutathione peroxidase 4 |
| 56637 | [*Gsk3b*](http://www.ncbi.nlm.nih.gov/sites/entrez?Db=gene&Cmd=ShowDetailView&TermToSearch=56637) | glycogen synthase kinase 3 beta |
| 103978 | [*Gpc5*](http://www.ncbi.nlm.nih.gov/sites/entrez?Db=gene&Cmd=ShowDetailView&TermToSearch=103978) | glypican 5 |
| 14683 | [*Gnas*](http://www.ncbi.nlm.nih.gov/sites/entrez?Db=gene&Cmd=ShowDetailView&TermToSearch=14683) | GNAS (guanine nucleotide binding protein, alpha stimulating) complex locus |
| 99412 | [*Golga2*](http://www.ncbi.nlm.nih.gov/sites/entrez?Db=gene&Cmd=ShowDetailView&TermToSearch=99412) | golgi autoantigen, golgin subfamily a, 2 |
| 14939 | [*Gzmb*](http://www.ncbi.nlm.nih.gov/sites/entrez?Db=gene&Cmd=ShowDetailView&TermToSearch=14939) | granzyme B |
| 14528 | [*Gch1*](http://www.ncbi.nlm.nih.gov/sites/entrez?Db=gene&Cmd=ShowDetailView&TermToSearch=14528) | GTP cyclohydrolase 1 |
| 13972 | [*Gnb1l*](http://www.ncbi.nlm.nih.gov/sites/entrez?Db=gene&Cmd=ShowDetailView&TermToSearch=13972) | guanine nucleotide binding protein (G protein), beta polypeptide 1-like |
| 14680 | [*Gnal*](http://www.ncbi.nlm.nih.gov/sites/entrez?Db=gene&Cmd=ShowDetailView&TermToSearch=14680) | guanine nucleotide binding protein, alpha stimulating, olfactory type |
| 14687 | [*Gnaz*](http://www.ncbi.nlm.nih.gov/sites/entrez?Db=gene&Cmd=ShowDetailView&TermToSearch=14687) | guanine nucleotide binding protein, alpha z subunit |
| 15439 | [*Hp*](http://www.ncbi.nlm.nih.gov/sites/entrez?Db=gene&Cmd=ShowDetailView&TermToSearch=15439) | haptoglobin |
| 14828 | [*Hspa5*](http://www.ncbi.nlm.nih.gov/sites/entrez?Db=gene&Cmd=ShowDetailView&TermToSearch=14828) | heat shock protein 5 |
| 22027 | [*Hsp90b1*](http://www.ncbi.nlm.nih.gov/sites/entrez?Db=gene&Cmd=ShowDetailView&TermToSearch=22027) | heat shock protein 90, beta (Grp94), member 1 |
| 14968* (100504404) | [*H2-Ea-ps*](https://www.ncbi.nlm.nih.gov/gene/100504404) | histocompatibility 2, class II antigen E alpha, pseudogene |
| 433759 | [*Hdac1*](http://www.ncbi.nlm.nih.gov/sites/entrez?Db=gene&Cmd=ShowDetailView&TermToSearch=433759) | histone deacetylase 1 |
| 26557 | [*Homer2*](http://www.ncbi.nlm.nih.gov/sites/entrez?Db=gene&Cmd=ShowDetailView&TermToSearch=26557) | homer homolog 2 (Drosophila) |
| 29816 | [*Hip1r*](http://www.ncbi.nlm.nih.gov/sites/entrez?Db=gene&Cmd=ShowDetailView&TermToSearch=29816) | huntingtin interacting protein 1 related |
| 76614 | [*Immt*](http://www.ncbi.nlm.nih.gov/sites/entrez?Db=gene&Cmd=ShowDetailView&TermToSearch=76614) | inner membrane protein, mitochondrial |
| 55980 | [*Impa1*](http://www.ncbi.nlm.nih.gov/sites/entrez?Db=gene&Cmd=ShowDetailView&TermToSearch=55980) | inositol (myo)-1(or 4)-monophosphatase 1 |
| 114663 | [*Impa2*](http://www.ncbi.nlm.nih.gov/sites/entrez?Db=gene&Cmd=ShowDetailView&TermToSearch=114663) | inositol (myo)-1(or 4)-monophosphatase 2 |
| 16438 | [*Itpr1*](http://www.ncbi.nlm.nih.gov/sites/entrez?Db=gene&Cmd=ShowDetailView&TermToSearch=16438) | inositol 1,4,5-triphosphate receptor 1 |
| 16329 | [*Inpp1*](http://www.ncbi.nlm.nih.gov/sites/entrez?Db=gene&Cmd=ShowDetailView&TermToSearch=16329) | inositol polyphosphate-1-phosphatase |
| 16000 | [*Igf1*](http://www.ncbi.nlm.nih.gov/sites/entrez?Db=gene&Cmd=ShowDetailView&TermToSearch=16000) | insulin-like growth factor 1 |
| 16008 | [*Igfbp2*](http://www.ncbi.nlm.nih.gov/sites/entrez?Db=gene&Cmd=ShowDetailView&TermToSearch=16008) | insulin-like growth factor binding protein 2 |
| 16176 | [*Il1b*](http://www.ncbi.nlm.nih.gov/sites/entrez?Db=gene&Cmd=ShowDetailView&TermToSearch=16176) | interleukin 1 beta |
| 16181 | [*Il1rn*](http://www.ncbi.nlm.nih.gov/sites/entrez?Db=gene&Cmd=ShowDetailView&TermToSearch=16181) | interleukin 1 receptor antagonist |
| 16173 | [*Il18*](http://www.ncbi.nlm.nih.gov/sites/entrez?Db=gene&Cmd=ShowDetailView&TermToSearch=16173) | interleukin 18 |
| 16185 | [*Il2rb*](http://www.ncbi.nlm.nih.gov/sites/entrez?Db=gene&Cmd=ShowDetailView&TermToSearch=16185) | interleukin 2 receptor, beta chain |
| 16193 | [*Il6*](http://www.ncbi.nlm.nih.gov/sites/entrez?Db=gene&Cmd=ShowDetailView&TermToSearch=16193) | interleukin 6 |
| 16199 | [*Il9r*](http://www.ncbi.nlm.nih.gov/sites/entrez?Db=gene&Cmd=ShowDetailView&TermToSearch=16199) | interleukin 9 receptor |
| 16443 | [*Itsn1*](http://www.ncbi.nlm.nih.gov/sites/entrez?Db=gene&Cmd=ShowDetailView&TermToSearch=16443) | intersectin 1 (SH3 domain protein 1A) |
| 16476 | [*Jun*](http://www.ncbi.nlm.nih.gov/sites/entrez?Db=gene&Cmd=ShowDetailView&TermToSearch=16476) | jun proto-oncogene |
| 545156 | [*Kalrn*](http://www.ncbi.nlm.nih.gov/sites/entrez?Db=gene&Cmd=ShowDetailView&TermToSearch=545156) | kalirin, RhoGEF kinase |
| 19144 | [*Klk6*](http://www.ncbi.nlm.nih.gov/sites/entrez?Db=gene&Cmd=ShowDetailView&TermToSearch=19144) | kallikrein related-peptidase 6 |
| 231912 | [*Katnal1*](http://www.ncbi.nlm.nih.gov/sites/entrez?Db=gene&Cmd=ShowDetailView&TermToSearch=231912) | katanin p60 subunit A-like 1 |
| 74187 | [*Katnb1*](http://www.ncbi.nlm.nih.gov/sites/entrez?Db=gene&Cmd=ShowDetailView&TermToSearch=74187) | katanin p80 (WD40-containing) subunit B 1 |
| 22439 | [*Xk*](http://www.ncbi.nlm.nih.gov/sites/entrez?Db=gene&Cmd=ShowDetailView&TermToSearch=22439) | Kell blood group precursor (McLeod phenotype) homolog |
| 102436 | [*Lars2*](http://www.ncbi.nlm.nih.gov/sites/entrez?Db=gene&Cmd=ShowDetailView&TermToSearch=102436) | leucyl-tRNA synthetase, mitochondrial |
| 108030 | [*Lin7a*](http://www.ncbi.nlm.nih.gov/sites/entrez?Db=gene&Cmd=ShowDetailView&TermToSearch=108030) | lin-7 homolog A (C. elegans) |
| 16842 | [*Lef1*](http://www.ncbi.nlm.nih.gov/sites/entrez?Db=gene&Cmd=ShowDetailView&TermToSearch=16842) | lymphoid enhancer binding factor 1 |
| 16992 | [*Lta*](http://www.ncbi.nlm.nih.gov/sites/entrez?Db=gene&Cmd=ShowDetailView&TermToSearch=16992) | lymphotoxin A |
| 14745 | [*Lpar1*](http://www.ncbi.nlm.nih.gov/sites/entrez?Db=gene&Cmd=ShowDetailView&TermToSearch=14745) | lysophosphatidic acid receptor 1 |
| 107029 | [*Me2*](http://www.ncbi.nlm.nih.gov/sites/entrez?Db=gene&Cmd=ShowDetailView&TermToSearch=107029) | malic enzyme 2, NAD(+)-dependent, mitochondrial |
| 17309 | [*Mgat3*](http://www.ncbi.nlm.nih.gov/sites/entrez?Db=gene&Cmd=ShowDetailView&TermToSearch=17309) | mannoside acetylglucosaminyltransferase 3 |
| 170790 | [*Mlc1*](http://www.ncbi.nlm.nih.gov/sites/entrez?Db=gene&Cmd=ShowDetailView&TermToSearch=170790) | megalencephalic leukoencephalopathy with subcortical cysts 1 homolog (human) |
| 17203 | [*Mc5r*](http://www.ncbi.nlm.nih.gov/sites/entrez?Db=gene&Cmd=ShowDetailView&TermToSearch=17203) | melanocortin 5 receptor |
| 108156 | [*Mthfd1*](http://www.ncbi.nlm.nih.gov/sites/entrez?Db=gene&Cmd=ShowDetailView&TermToSearch=108156) | methylenetetrahydrofolate dehydrogenase (NADP+ dependent), methenyltetrahydrofolate cyclohydrolase, formyltetrahydrofolate synthase |
| 17754 | [*Map1a*](http://www.ncbi.nlm.nih.gov/sites/entrez?Db=gene&Cmd=ShowDetailView&TermToSearch=17754) | microtubule-associated protein 1 A |
| 26413 | [*Mapk1*](http://www.ncbi.nlm.nih.gov/sites/entrez?Db=gene&Cmd=ShowDetailView&TermToSearch=26413) | mitogen-activated protein kinase 1 |
| 26417 | [*Mapk3*](http://www.ncbi.nlm.nih.gov/sites/entrez?Db=gene&Cmd=ShowDetailView&TermToSearch=26417) | mitogen-activated protein kinase 3 |
| 17161 | [*Maoa*](http://www.ncbi.nlm.nih.gov/sites/entrez?Db=gene&Cmd=ShowDetailView&TermToSearch=17161) | monoamine oxidase A |
| 17153 | [*Mal*](http://www.ncbi.nlm.nih.gov/sites/entrez?Db=gene&Cmd=ShowDetailView&TermToSearch=17153) | myelin and lymphocyte protein, T cell differentiation protein |
| 17196 | [*Mbp*](http://www.ncbi.nlm.nih.gov/sites/entrez?Db=gene&Cmd=ShowDetailView&TermToSearch=17196) | myelin basic protein |
| 17441 | [*Mog*](http://www.ncbi.nlm.nih.gov/sites/entrez?Db=gene&Cmd=ShowDetailView&TermToSearch=17441) | myelin oligodendrocyte glycoprotein |
| 17136 | [*Mag*](http://www.ncbi.nlm.nih.gov/sites/entrez?Db=gene&Cmd=ShowDetailView&TermToSearch=17136) | myelin-associated glycoprotein |
| 17433 | [*Mobp*](http://www.ncbi.nlm.nih.gov/sites/entrez?Db=gene&Cmd=ShowDetailView&TermToSearch=17433) | myelin-associated oligodendrocytic basic protein |
| 17869 | [*Myc*](http://www.ncbi.nlm.nih.gov/sites/entrez?Db=gene&Cmd=ShowDetailView&TermToSearch=17869) | myelocytomatosis oncogene |
| 71780 | [*Isyna1*](http://www.ncbi.nlm.nih.gov/sites/entrez?Db=gene&Cmd=ShowDetailView&TermToSearch=71780) | myo-inositol 1-phosphate synthase A1 |
| 108123 | [*Napg*](http://www.ncbi.nlm.nih.gov/sites/entrez?Db=gene&Cmd=ShowDetailView&TermToSearch=108123) | N-ethylmaleimide sensitive fusion protein attachment protein gamma |
| 75406 | [*Ndufs7*](http://www.ncbi.nlm.nih.gov/sites/entrez?Db=gene&Cmd=ShowDetailView&TermToSearch=75406) | NADH dehydrogenase (ubiquinone) Fe-S protein 7 |
| 225887 | [*Ndufs8*](http://www.ncbi.nlm.nih.gov/sites/entrez?Db=gene&Cmd=ShowDetailView&TermToSearch=225887) | NADH dehydrogenase (ubiquinone) Fe-S protein 8 |
| 17995 | [*Ndufv1*](http://www.ncbi.nlm.nih.gov/sites/entrez?Db=gene&Cmd=ShowDetailView&TermToSearch=17995) | NADH dehydrogenase (ubiquinone) flavoprotein 1 |
| 72900 | [*Ndufv2*](http://www.ncbi.nlm.nih.gov/sites/entrez?Db=gene&Cmd=ShowDetailView&TermToSearch=72900) | NADH dehydrogenase (ubiquinone) flavoprotein 2 |
| 17719 | [*ND4*](http://www.ncbi.nlm.nih.gov/sites/entrez?Db=gene&Cmd=ShowDetailView&TermToSearch=17719) | NADH dehydrogenase subunit 4 |
| 17967 | [*Ncam1*](http://www.ncbi.nlm.nih.gov/sites/entrez?Db=gene&Cmd=ShowDetailView&TermToSearch=17967) | neural cell adhesion molecule 1 |
| 83814 | [*Nedd4l*](http://www.ncbi.nlm.nih.gov/sites/entrez?Db=gene&Cmd=ShowDetailView&TermToSearch=83814) | neural precursor cell expressed, developmentally down-regulated gene 4-like |
| 211323 | [*Nrg1*](http://www.ncbi.nlm.nih.gov/sites/entrez?Db=gene&Cmd=ShowDetailView&TermToSearch=211323) | neuregulin 1 |
| 18143 | [*Npas2*](http://www.ncbi.nlm.nih.gov/sites/entrez?Db=gene&Cmd=ShowDetailView&TermToSearch=18143) | neuronal PAS domain protein 2 |
| 109648 | [*Npy*](http://www.ncbi.nlm.nih.gov/sites/entrez?Db=gene&Cmd=ShowDetailView&TermToSearch=109648) | neuropeptide Y |
| 18211 | [*Ntrk1*](http://www.ncbi.nlm.nih.gov/sites/entrez?Db=gene&Cmd=ShowDetailView&TermToSearch=18211) | neurotrophic tyrosine kinase, receptor, type 1 |
| 18212 | [*Ntrk2*](http://www.ncbi.nlm.nih.gov/sites/entrez?Db=gene&Cmd=ShowDetailView&TermToSearch=18212) | neurotrophic tyrosine kinase, receptor, type 2 |
| 18205 | [*Ntf3*](http://www.ncbi.nlm.nih.gov/sites/entrez?Db=gene&Cmd=ShowDetailView&TermToSearch=18205) | neurotrophin 3 |
| 70729 | [*Nos1ap*](http://www.ncbi.nlm.nih.gov/sites/entrez?Db=gene&Cmd=ShowDetailView&TermToSearch=70729) | nitric oxide synthase 1 (neuronal) adaptor protein |
| 18125 | [*Nos1*](http://www.ncbi.nlm.nih.gov/sites/entrez?Db=gene&Cmd=ShowDetailView&TermToSearch=18125) | nitric oxide synthase 1, neuronal |
| 18127 | [*Nos3*](http://www.ncbi.nlm.nih.gov/sites/entrez?Db=gene&Cmd=ShowDetailView&TermToSearch=18127) | nitric oxide synthase 3, endothelial cell |
| 67203 | [*Nde1*](http://www.ncbi.nlm.nih.gov/sites/entrez?Db=gene&Cmd=ShowDetailView&TermToSearch=67203) | nudE neurodevelopmental protein 1 |
| 83431 | [*Ndel1*](http://www.ncbi.nlm.nih.gov/sites/entrez?Db=gene&Cmd=ShowDetailView&TermToSearch=83431) | nudE neurodevelopmental protein 1 like 1 |
| 18033 | [*Nfkb1*](http://www.ncbi.nlm.nih.gov/sites/entrez?Db=gene&Cmd=ShowDetailView&TermToSearch=18033) | nuclear factor of kappa light polypeptide gene enhancer in B cells 1, p105 |
| 18034 | [*Nfkb2*](http://www.ncbi.nlm.nih.gov/sites/entrez?Db=gene&Cmd=ShowDetailView&TermToSearch=18034) | nuclear factor of kappa light polypeptide gene enhancer in B cells 2, p49/p100 |
| 18036 | [*Nfkbib*](http://www.ncbi.nlm.nih.gov/sites/entrez?Db=gene&Cmd=ShowDetailView&TermToSearch=18036) | nuclear factor of kappa light polypeptide gene enhancer in B cells inhibitor, beta |
| 217166 | [*Nr1d1*](http://www.ncbi.nlm.nih.gov/sites/entrez?Db=gene&Cmd=ShowDetailView&TermToSearch=217166) | nuclear receptor subfamily 1, group D, member 1 |
| 14815 | [*Nr3c1*](http://www.ncbi.nlm.nih.gov/sites/entrez?Db=gene&Cmd=ShowDetailView&TermToSearch=14815) | nuclear receptor subfamily 3, group C, member 1 |
| 71207 | [*Nudt4*](http://www.ncbi.nlm.nih.gov/sites/entrez?Db=gene&Cmd=ShowDetailView&TermToSearch=71207) | nudix (nucleoside diphosphate linked moiety X)-type motif 4 |
| 50914 | [*Olig1*](http://www.ncbi.nlm.nih.gov/sites/entrez?Db=gene&Cmd=ShowDetailView&TermToSearch=50914) | oligodendrocyte transcription factor 1 |
| 50913 | [*Olig2*](http://www.ncbi.nlm.nih.gov/sites/entrez?Db=gene&Cmd=ShowDetailView&TermToSearch=50913) | oligodendrocyte transcription factor 2 |
| 18424 | [*Otx2*](http://www.ncbi.nlm.nih.gov/sites/entrez?Db=gene&Cmd=ShowDetailView&TermToSearch=18424) | orthodenticle homolog 2 |
| 18507 | [*Pax5*](http://www.ncbi.nlm.nih.gov/sites/entrez?Db=gene&Cmd=ShowDetailView&TermToSearch=18507) | paired box gene 5 |
| 56376 | [*Pdlim5*](http://www.ncbi.nlm.nih.gov/sites/entrez?Db=gene&Cmd=ShowDetailView&TermToSearch=56376) | PDZ and LIM domain 5 |
| 56031 | [*Ppie*](http://www.ncbi.nlm.nih.gov/sites/entrez?Db=gene&Cmd=ShowDetailView&TermToSearch=56031) | peptidylprolyl isomerase E (cyclophilin E) |
| 18646 | [*Prf1*](http://www.ncbi.nlm.nih.gov/sites/entrez?Db=gene&Cmd=ShowDetailView&TermToSearch=18646) | perforin 1 (pore forming protein) |
| 18541 | [*Pcnt*](http://www.ncbi.nlm.nih.gov/sites/entrez?Db=gene&Cmd=ShowDetailView&TermToSearch=18541) | pericentrin (kendrin) |
| 18626 | [*Per1*](http://www.ncbi.nlm.nih.gov/sites/entrez?Db=gene&Cmd=ShowDetailView&TermToSearch=18626) | period circadian clock 1 |
| 18627 | [*Per2*](http://www.ncbi.nlm.nih.gov/sites/entrez?Db=gene&Cmd=ShowDetailView&TermToSearch=18627) | period circadian clock 2 |
| 18628 | [*Per3*](http://www.ncbi.nlm.nih.gov/sites/entrez?Db=gene&Cmd=ShowDetailView&TermToSearch=18628) | period circadian clock 3 |
| 18858 | [*Pmp22*](http://www.ncbi.nlm.nih.gov/sites/entrez?Db=gene&Cmd=ShowDetailView&TermToSearch=18858) | peripheral myelin protein 22 |
| 224020 | [*Pi4ka*](http://www.ncbi.nlm.nih.gov/sites/entrez?Db=gene&Cmd=ShowDetailView&TermToSearch=224020) | phosphatidylinositol 4-kinase, catalytic, alpha polypeptide |
| 107650 | [*Pi4kb*](http://www.ncbi.nlm.nih.gov/sites/entrez?Db=gene&Cmd=ShowDetailView&TermToSearch=107650) | phosphatidylinositol 4-kinase, catalytic, beta polypeptide |
| 56305 | [*Pitpnb*](http://www.ncbi.nlm.nih.gov/sites/entrez?Db=gene&Cmd=ShowDetailView&TermToSearch=56305) | phosphatidylinositol transfer protein, beta |
| 18718 | [*Pip4k2a*](http://www.ncbi.nlm.nih.gov/sites/entrez?Db=gene&Cmd=ShowDetailView&TermToSearch=18718) | phosphatidylinositol-5-phosphate 4-kinase, type II, alpha |
| 18578 | [*Pde4b*](http://www.ncbi.nlm.nih.gov/sites/entrez?Db=gene&Cmd=ShowDetailView&TermToSearch=18578) | phosphodiesterase 4B, cAMP specific |
| 238871 | [*Pde4d*](http://www.ncbi.nlm.nih.gov/sites/entrez?Db=gene&Cmd=ShowDetailView&TermToSearch=238871) | phosphodiesterase 4D, cAMP specific |
| 18655 | [*Pgk1*](http://www.ncbi.nlm.nih.gov/sites/entrez?Db=gene&Cmd=ShowDetailView&TermToSearch=18655) | phosphoglycerate kinase 1 |
| 225326 | [*Pik3c3*](http://www.ncbi.nlm.nih.gov/sites/entrez?Db=gene&Cmd=ShowDetailView&TermToSearch=225326) | phosphoinositide-3-kinase, class 3 |
| 18708 | [*Pik3r1*](http://www.ncbi.nlm.nih.gov/sites/entrez?Db=gene&Cmd=ShowDetailView&TermToSearch=18708) | phosphatidylinositol 3-kinase, regulatory subunit, polypeptide 1 (p85 alpha) |
| 18778 | [*Pla2g1b*](http://www.ncbi.nlm.nih.gov/sites/entrez?Db=gene&Cmd=ShowDetailView&TermToSearch=18778) | phospholipase A2, group IB, pancreas |
| 18780 | [*Pla2g2a*](http://www.ncbi.nlm.nih.gov/sites/entrez?Db=gene&Cmd=ShowDetailView&TermToSearch=18780) | phospholipase A2, group IIA (platelets, synovial fluid) |
| 237625 | [*Pla2g3*](http://www.ncbi.nlm.nih.gov/sites/entrez?Db=gene&Cmd=ShowDetailView&TermToSearch=237625) | phospholipase A2, group III |
| 18783 | [*Pla2g4a*](http://www.ncbi.nlm.nih.gov/sites/entrez?Db=gene&Cmd=ShowDetailView&TermToSearch=18783) | phospholipase A2, group IVA (cytosolic, calcium-dependent) |
| 211429 | [*Pla2g4b*](http://www.ncbi.nlm.nih.gov/sites/entrez?Db=gene&Cmd=ShowDetailView&TermToSearch=211429) | phospholipase A2, group IVB (cytosolic) |
| 232889 | [*Pla2g4c*](http://www.ncbi.nlm.nih.gov/sites/entrez?Db=gene&Cmd=ShowDetailView&TermToSearch=232889) | phospholipase A2, group IVC (cytosolic, calcium-independent) |
| 78390 | [*Pla2g4d*](http://www.ncbi.nlm.nih.gov/sites/entrez?Db=gene&Cmd=ShowDetailView&TermToSearch=78390) | phospholipase A2, group IVD |
| 18784 | [*Pla2g5*](http://www.ncbi.nlm.nih.gov/sites/entrez?Db=gene&Cmd=ShowDetailView&TermToSearch=18784) | phospholipase A2, group V |
| 18803 | [*Plcg1*](http://www.ncbi.nlm.nih.gov/sites/entrez?Db=gene&Cmd=ShowDetailView&TermToSearch=18803) | phospholipase C, gamma 1 |
| 235527 | [*Plscr4*](http://www.ncbi.nlm.nih.gov/sites/entrez?Db=gene&Cmd=ShowDetailView&TermToSearch=235527) | phospholipid scramblase 4 |
| 18472 | [*Pafah1b1*](http://www.ncbi.nlm.nih.gov/sites/entrez?Db=gene&Cmd=ShowDetailView&TermToSearch=18472) | platelet-activating factor acetylhydrolase, isoform 1b, subunit 1 |
| 18476 | [*Pafah1b3*](http://www.ncbi.nlm.nih.gov/sites/entrez?Db=gene&Cmd=ShowDetailView&TermToSearch=18476) | platelet-activating factor acetylhydrolase, isoform 1b, subunit 3 |
| 11545 | [*Parp1*](http://www.ncbi.nlm.nih.gov/sites/entrez?Db=gene&Cmd=ShowDetailView&TermToSearch=11545) | poly (ADP-ribose) polymerase family, member 1 |
| 22658 | [*Pcgf2*](http://www.ncbi.nlm.nih.gov/sites/entrez?Db=gene&Cmd=ShowDetailView&TermToSearch=22658) | polycomb group ring finger 2 |
| 18975 | [*Polg*](http://www.ncbi.nlm.nih.gov/sites/entrez?Db=gene&Cmd=ShowDetailView&TermToSearch=18975) | polymerase (DNA directed), gamma |
| 16525 | [*Kcnk1*](http://www.ncbi.nlm.nih.gov/sites/entrez?Db=gene&Cmd=ShowDetailView&TermToSearch=16525) | potassium channel, subfamily K, member 1 |
| 140493 | [*Kcnn3*](http://www.ncbi.nlm.nih.gov/sites/entrez?Db=gene&Cmd=ShowDetailView&TermToSearch=140493) | potassium intermediate/small conductance calcium-activated channel, subfamily N, member 3 |
| 16536 | [*Kcnq2*](http://www.ncbi.nlm.nih.gov/sites/entrez?Db=gene&Cmd=ShowDetailView&TermToSearch=16536) | potassium voltage-gated channel, subfamily Q, member 2 |
| 226922 | [*Kcnq5*](http://www.ncbi.nlm.nih.gov/sites/entrez?Db=gene&Cmd=ShowDetailView&TermToSearch=226922) | potassium voltage-gated channel, subfamily Q, member 5 |
| 18619 | [*Penk*](http://www.ncbi.nlm.nih.gov/sites/entrez?Db=gene&Cmd=ShowDetailView&TermToSearch=18619) | preproenkephalin |
| 114774 | [*Pawr*](http://www.ncbi.nlm.nih.gov/sites/entrez?Db=gene&Cmd=ShowDetailView&TermToSearch=114774) | PRKC, apoptosis, WT1, regulator |
| 110312 | [*Pmch*](http://www.ncbi.nlm.nih.gov/sites/entrez?Db=gene&Cmd=ShowDetailView&TermToSearch=110312) | pro-melanin-concentrating hormone |
| 18645 | [*Pfn2*](http://www.ncbi.nlm.nih.gov/sites/entrez?Db=gene&Cmd=ShowDetailView&TermToSearch=18645) | profilin 2 |
| 19072 | [*Prep*](http://www.ncbi.nlm.nih.gov/sites/entrez?Db=gene&Cmd=ShowDetailView&TermToSearch=19072) | prolyl endopeptidase |
| 19225 | [*Ptgs2*](http://www.ncbi.nlm.nih.gov/sites/entrez?Db=gene&Cmd=ShowDetailView&TermToSearch=19225) | prostaglandin-endoperoxide synthase 2 |
| 67089 | [*Psmc6*](http://www.ncbi.nlm.nih.gov/sites/entrez?Db=gene&Cmd=ShowDetailView&TermToSearch=67089) | proteasome (prosome, macropain) 26S subunit, ATPase, 6 |
| 57296 | [*Psmd8*](http://www.ncbi.nlm.nih.gov/sites/entrez?Db=gene&Cmd=ShowDetailView&TermToSearch=57296) | proteasome (prosome, macropain) 26S subunit, non-ATPase, 8 |
| 18750 | [*Prkca*](http://www.ncbi.nlm.nih.gov/sites/entrez?Db=gene&Cmd=ShowDetailView&TermToSearch=18750) | protein kinase C, alpha |
| 18751 | [*Prkcb*](http://www.ncbi.nlm.nih.gov/sites/entrez?Db=gene&Cmd=ShowDetailView&TermToSearch=18751) | protein kinase C, beta |
| 18753 | [*Prkcd*](http://www.ncbi.nlm.nih.gov/sites/entrez?Db=gene&Cmd=ShowDetailView&TermToSearch=18753) | protein kinase C, delta |
| 18754 | [*Prkce*](http://www.ncbi.nlm.nih.gov/sites/entrez?Db=gene&Cmd=ShowDetailView&TermToSearch=18754) | protein kinase C, epsilon |
| 18752 | [*Prkc*](http://www.ncbi.nlm.nih.gov/sites/entrez?Db=gene&Cmd=ShowDetailView&TermToSearch=18752)*g* | protein kinase C, gamma |
| 18759 | [*Prkci*](http://www.ncbi.nlm.nih.gov/sites/entrez?Db=gene&Cmd=ShowDetailView&TermToSearch=18759) | protein kinase C, iota |
| 18761 | [*Prkcq*](http://www.ncbi.nlm.nih.gov/sites/entrez?Db=gene&Cmd=ShowDetailView&TermToSearch=18761) | protein kinase C, theta |
| 18762 | [*Prkcz*](http://www.ncbi.nlm.nih.gov/sites/entrez?Db=gene&Cmd=ShowDetailView&TermToSearch=18762) | protein kinase C, zeta |
| 75292 | [*Prkd3*](http://www.ncbi.nlm.nih.gov/sites/entrez?Db=gene&Cmd=ShowDetailView&TermToSearch=75292) | protein kinase D3 |
| 18747 | [*Prkaca*](http://www.ncbi.nlm.nih.gov/sites/entrez?Db=gene&Cmd=ShowDetailView&TermToSearch=18747) | protein kinase, cAMP dependent, catalytic, alpha |
| 18749 | [*Prkacb*](http://www.ncbi.nlm.nih.gov/sites/entrez?Db=gene&Cmd=ShowDetailView&TermToSearch=18749) | protein kinase, cAMP dependent, catalytic, beta |
| 19049 | [*Ppp1r1b*](http://www.ncbi.nlm.nih.gov/sites/entrez?Db=gene&Cmd=ShowDetailView&TermToSearch=19049) | protein phosphatase 1, regulatory (inhibitor) subunit 1B |
| 269643 | [*Ppp2r2c*](http://www.ncbi.nlm.nih.gov/sites/entrez?Db=gene&Cmd=ShowDetailView&TermToSearch=269643) | protein phosphatase 2, regulatory subunit B, gamma |
| 26931 | [*Ppp2r5c*](http://www.ncbi.nlm.nih.gov/sites/entrez?Db=gene&Cmd=ShowDetailView&TermToSearch=26931) | protein phosphatase 2, regulatory subunit B´, gamma |
| 68507 | [*Ppfia4*](http://www.ncbi.nlm.nih.gov/sites/entrez?Db=gene&Cmd=ShowDetailView&TermToSearch=68507) | protein tyrosine phosphatase, receptor type, f polypeptide (PTPRF), interacting protein (liprin), alpha 4 |
| 18823 | [*Plp1*](http://www.ncbi.nlm.nih.gov/sites/entrez?Db=gene&Cmd=ShowDetailView&TermToSearch=18823) | proteolipid protein (myelin) 1 |
| 19290 | [*Pura*](http://www.ncbi.nlm.nih.gov/sites/entrez?Db=gene&Cmd=ShowDetailView&TermToSearch=19290) | purine rich element binding protein A |
| 18439 | [*P2rx7*](http://www.ncbi.nlm.nih.gov/sites/entrez?Db=gene&Cmd=ShowDetailView&TermToSearch=18439) | purinergic receptor P2X, ligand-gated ion channel, 7 |
| 19349 | [*Rab7*](http://www.ncbi.nlm.nih.gov/sites/entrez?Db=gene&Cmd=ShowDetailView&TermToSearch=19349) | RAB7, member RAS oncogene family |
| 56705 | [*Ranbp9*](http://www.ncbi.nlm.nih.gov/sites/entrez?Db=gene&Cmd=ShowDetailView&TermToSearch=56705) | RAN binding protein 9 |
| 110351 | [*Rap1gap*](http://www.ncbi.nlm.nih.gov/sites/entrez?Db=gene&Cmd=ShowDetailView&TermToSearch=110351) | Rap1 GTPase-activating protein |
| 19766 | [*Ripk1*](http://www.ncbi.nlm.nih.gov/sites/entrez?Db=gene&Cmd=ShowDetailView&TermToSearch=19766) | receptor (TNFRSF)-interacting serine-threonine kinase 1 |
| 19699 | [*Reln*](http://www.ncbi.nlm.nih.gov/sites/entrez?Db=gene&Cmd=ShowDetailView&TermToSearch=19699) | reelin |
| 18489 | [*Reg3b*](http://www.ncbi.nlm.nih.gov/sites/entrez?Db=gene&Cmd=ShowDetailView&TermToSearch=18489) | regenerating islet-derived 3 beta |
| 19736 | [*Rgs4*](http://www.ncbi.nlm.nih.gov/sites/entrez?Db=gene&Cmd=ShowDetailView&TermToSearch=19736) | regulator of G-protein signaling 4 |
| 71137 | [*Rfx4*](http://www.ncbi.nlm.nih.gov/sites/entrez?Db=gene&Cmd=ShowDetailView&TermToSearch=71137) | regulatory factor X, 4 (influences HLA class II expression) |
| 68585 | [*Rtn4*](http://www.ncbi.nlm.nih.gov/sites/entrez?Db=gene&Cmd=ShowDetailView&TermToSearch=68585) | reticulon 4 |
| 19821 | [*Rnf2*](http://www.ncbi.nlm.nih.gov/sites/entrez?Db=gene&Cmd=ShowDetailView&TermToSearch=19821) | ring finger protein 2 |
| 12393 | [*Runx2*](http://www.ncbi.nlm.nih.gov/sites/entrez?Db=gene&Cmd=ShowDetailView&TermToSearch=12393) | runt related transcription factor 2 |
| 11702 | [*Amd1*](http://www.ncbi.nlm.nih.gov/sites/entrez?Db=gene&Cmd=ShowDetailView&TermToSearch=11702) | S-adenosylmethionine decarboxylase 1 |
| 20202 | [*S100a9*](http://www.ncbi.nlm.nih.gov/sites/entrez?Db=gene&Cmd=ShowDetailView&TermToSearch=20202) | S100 calcium binding protein A9 (calgranulin B) |
| 353282 | [*Sfmbt2*](http://www.ncbi.nlm.nih.gov/sites/entrez?Db=gene&Cmd=ShowDetailView&TermToSearch=353282) | Scm-like with four mbt domains 2 |
| 20254 | [*Scg2*](http://www.ncbi.nlm.nih.gov/sites/entrez?Db=gene&Cmd=ShowDetailView&TermToSearch=20254) | secretogranin II |
| 20751 | [*Spr*](http://www.ncbi.nlm.nih.gov/sites/entrez?Db=gene&Cmd=ShowDetailView&TermToSearch=20751) | sepiapterin reductase |
| 107723 | [*Slc12a6*](http://www.ncbi.nlm.nih.gov/sites/entrez?Db=gene&Cmd=ShowDetailView&TermToSearch=107723) | solute carrier family 12, member 6 |
| 237831 | [*Slc13a5*](http://www.ncbi.nlm.nih.gov/sites/entrez?Db=gene&Cmd=ShowDetailView&TermToSearch=237831) | solute carrier family 13 (sodium-dependent citrate transporter), member 5 |
| 56643 | [*Slc15a1*](http://www.ncbi.nlm.nih.gov/sites/entrez?Db=gene&Cmd=ShowDetailView&TermToSearch=56643) | solute carrier family 15 (oligopeptide transporter), member 1 |
| 110877 | [*Slc18a1*](http://www.ncbi.nlm.nih.gov/sites/entrez?Db=gene&Cmd=ShowDetailView&TermToSearch=110877) | solute carrier family 18 (vesicular monoamine), member 1 |
| 214084 | [*Slc18a2*](http://www.ncbi.nlm.nih.gov/sites/entrez?Db=gene&Cmd=ShowDetailView&TermToSearch=214084) | solute carrier family 18 (vesicular monoamine), member 2 |
| 53881 | [*Slc5a3*](http://www.ncbi.nlm.nih.gov/sites/entrez?Db=gene&Cmd=ShowDetailView&TermToSearch=53881) | solute carrier family 5 (inositol transporters), member 3 |
| 13162 | [*Slc6a3*](http://www.ncbi.nlm.nih.gov/sites/entrez?Db=gene&Cmd=ShowDetailView&TermToSearch=13162) | solute carrier family 6 (neurotransmitter transporter, dopamine), member 3 |
| 20538 | [*Slc6a2*](http://www.ncbi.nlm.nih.gov/sites/entrez?Db=gene&Cmd=ShowDetailView&TermToSearch=20538) | solute carrier family 6 (neurotransmitter transporter, noradrenalin), member 2 |
| 15567 | [*Slc6a4*](http://www.ncbi.nlm.nih.gov/sites/entrez?Db=gene&Cmd=ShowDetailView&TermToSearch=15567) | solute carrier family 6 (neurotransmitter transporter, serotonin), member 4 |
| 20604 | [*Sst*](http://www.ncbi.nlm.nih.gov/sites/entrez?Db=gene&Cmd=ShowDetailView&TermToSearch=20604) | somatostatin |
| 20740 | [*Sptan1*](http://www.ncbi.nlm.nih.gov/sites/entrez?Db=gene&Cmd=ShowDetailView&TermToSearch=20740) | spectrin alpha, non-erythrocytic 1 |
| 80297 | [*Sptbn4*](http://www.ncbi.nlm.nih.gov/sites/entrez?Db=gene&Cmd=ShowDetailView&TermToSearch=80297) | spectrin beta, non-erythrocytic 4 |
| 20229 | [*Sat1*](http://www.ncbi.nlm.nih.gov/sites/entrez?Db=gene&Cmd=ShowDetailView&TermToSearch=20229) | spermidine/spermine N1-acetyl transferase 1 |
| 20665 | [*Sox10*](http://www.ncbi.nlm.nih.gov/sites/entrez?Db=gene&Cmd=ShowDetailView&TermToSearch=20665) | SRY (sex determining region Y)-box 10 |
| 20450 | [*St8sia2*](http://www.ncbi.nlm.nih.gov/sites/entrez?Db=gene&Cmd=ShowDetailView&TermToSearch=20450) | ST8 alpha-N-acetyl-neuraminide alpha-2,8-sialyltransferase 2 |
| 20845 | [*Star*](http://www.ncbi.nlm.nih.gov/sites/entrez?Db=gene&Cmd=ShowDetailView&TermToSearch=20845) | steroidogenic acute regulatory protein |
| 70099 | [*Smc4*](http://www.ncbi.nlm.nih.gov/sites/entrez?Db=gene&Cmd=ShowDetailView&TermToSearch=70099) | structural maintenance of chromosomes 4 |
| 20887 | [*Sult1a1*](http://www.ncbi.nlm.nih.gov/sites/entrez?Db=gene&Cmd=ShowDetailView&TermToSearch=20887) | sulfotransferase family 1A, phenol-preferring, member 1 |
| 20655 | [*Sod1*](http://www.ncbi.nlm.nih.gov/sites/entrez?Db=gene&Cmd=ShowDetailView&TermToSearch=20655) | superoxide dismutase 1, soluble |
| 57376 | [*Smarce1*](http://www.ncbi.nlm.nih.gov/sites/entrez?Db=gene&Cmd=ShowDetailView&TermToSearch=57376) | SWI/SNF related, matrix associated, actin dependent regulator of chromatin, subfamily e, member 1 |
| 27204 | [*Syn3*](http://www.ncbi.nlm.nih.gov/sites/entrez?Db=gene&Cmd=ShowDetailView&TermToSearch=27204) | synapsin III |
| 64009 | [*Syne1*](http://www.ncbi.nlm.nih.gov/sites/entrez?Db=gene&Cmd=ShowDetailView&TermToSearch=64009) | synaptic nuclear envelope 1 |
| 20972 | [*Syngr1*](http://www.ncbi.nlm.nih.gov/sites/entrez?Db=gene&Cmd=ShowDetailView&TermToSearch=20972) | synaptogyrin 1 |
| 104015 | [*Synj1*](http://www.ncbi.nlm.nih.gov/sites/entrez?Db=gene&Cmd=ShowDetailView&TermToSearch=104015) | synaptojanin 1 |
| 67474 | [*Snap29*](http://www.ncbi.nlm.nih.gov/sites/entrez?Db=gene&Cmd=ShowDetailView&TermToSearch=67474) | synaptosomal-associated protein 29 |
| 21380 | [*Tbx1*](http://www.ncbi.nlm.nih.gov/sites/entrez?Db=gene&Cmd=ShowDetailView&TermToSearch=21380) | T-box 1 |
| 21815 | [*Tgif1*](http://www.ncbi.nlm.nih.gov/sites/entrez?Db=gene&Cmd=ShowDetailView&TermToSearch=21815) | TGFB-induced factor homeobox 1 |
| 11651 | [*Akt1*](http://www.ncbi.nlm.nih.gov/sites/entrez?Db=gene&Cmd=ShowDetailView&TermToSearch=11651) | thymoma viral proto-oncogene 1 |
| 21833 | [*Thra*](http://www.ncbi.nlm.nih.gov/sites/entrez?Db=gene&Cmd=ShowDetailView&TermToSearch=21833) | thyroid hormone receptor alpha |
| 14897 | [*Trip12*](http://www.ncbi.nlm.nih.gov/sites/entrez?Db=gene&Cmd=ShowDetailView&TermToSearch=14897) | thyroid hormone receptor interactor 12 |
| 21853 | [*Timeless*](http://www.ncbi.nlm.nih.gov/sites/entrez?Db=gene&Cmd=ShowDetailView&TermToSearch=21853) | timeless circadian clock 1 |
| 22029 | [*Traf1*](http://www.ncbi.nlm.nih.gov/sites/entrez?Db=gene&Cmd=ShowDetailView&TermToSearch=22029) | TNF receptor-associated factor 1 |
| 30931 | [*Tor1a*](http://www.ncbi.nlm.nih.gov/sites/entrez?Db=gene&Cmd=ShowDetailView&TermToSearch=30931) | torsin family 1, member A (torsin A) |
| 215855 | [*Taar6*](http://www.ncbi.nlm.nih.gov/sites/entrez?Db=gene&Cmd=ShowDetailView&TermToSearch=215855) | trace amine-associated receptor 6 |
| 74019 | [*Traf3ip1*](http://www.ncbi.nlm.nih.gov/sites/entrez?Db=gene&Cmd=ShowDetailView&TermToSearch=74019) | TRAF3 interacting protein 1 |
| 21413 | [*Tcf4*](http://www.ncbi.nlm.nih.gov/sites/entrez?Db=gene&Cmd=ShowDetailView&TermToSearch=21413) | transcription factor 4 |
| 21414 | [*Tcf7*](http://www.ncbi.nlm.nih.gov/sites/entrez?Db=gene&Cmd=ShowDetailView&TermToSearch=21414) | transcription factor 7, T cell specific |
| 21419 | [*Tfap2b*](http://www.ncbi.nlm.nih.gov/sites/entrez?Db=gene&Cmd=ShowDetailView&TermToSearch=21419) | transcription factor AP-2 beta |
| 21418 | [*Tfap2a*](http://www.ncbi.nlm.nih.gov/sites/entrez?Db=gene&Cmd=ShowDetailView&TermToSearch=21418) | transcription factor AP-2, alpha |
| 21885 | [*Tle1*](http://www.ncbi.nlm.nih.gov/sites/entrez?Db=gene&Cmd=ShowDetailView&TermToSearch=21885) | transducin-like enhancer of split 1, homolog of Drosophila E(spl) |
| 22041 | [*Trf*](http://www.ncbi.nlm.nih.gov/sites/entrez?Db=gene&Cmd=ShowDetailView&TermToSearch=22041) | transferrin |
| 21803 | [*Tgfb1*](http://www.ncbi.nlm.nih.gov/sites/entrez?Db=gene&Cmd=ShowDetailView&TermToSearch=21803) | transforming growth factor, beta 1 |
| 26946 | [*Trpc7*](http://www.ncbi.nlm.nih.gov/sites/entrez?Db=gene&Cmd=ShowDetailView&TermToSearch=26946) | transient receptor potential cation channel, subfamily C, member 7 |
| 28240 | [*Trpm2*](http://www.ncbi.nlm.nih.gov/sites/entrez?Db=gene&Cmd=ShowDetailView&TermToSearch=28240) | transient receptor potential cation channel, subfamily M, member 2 |
| 64177 | [*Trpv6*](http://www.ncbi.nlm.nih.gov/sites/entrez?Db=gene&Cmd=ShowDetailView&TermToSearch=64177) | transient receptor potential cation channel, subfamily V, member 6 |
| 53424 | [*Tsnax*](http://www.ncbi.nlm.nih.gov/sites/entrez?Db=gene&Cmd=ShowDetailView&TermToSearch=53424) | translin-associated factor X |
| 21955 | [*Tnnt1*](http://www.ncbi.nlm.nih.gov/sites/entrez?Db=gene&Cmd=ShowDetailView&TermToSearch=21955) | troponin T1, skeletal, slow |
| 21990 | [*Tph1*](http://www.ncbi.nlm.nih.gov/sites/entrez?Db=gene&Cmd=ShowDetailView&TermToSearch=21990) | tryptophan hydroxylase 1 |
| 216343 | [*Tph2*](http://www.ncbi.nlm.nih.gov/sites/entrez?Db=gene&Cmd=ShowDetailView&TermToSearch=216343) | tryptophan hydroxylase 2 |
| 53857 | [*Tuba8*](http://www.ncbi.nlm.nih.gov/sites/entrez?Db=gene&Cmd=ShowDetailView&TermToSearch=53857) | tubulin, alpha 8 |
| 21926 | [*Tnf*](http://www.ncbi.nlm.nih.gov/sites/entrez?Db=gene&Cmd=ShowDetailView&TermToSearch=21926) | tumor necrosis factor |
| 22629 | [*Ywhah*](http://www.ncbi.nlm.nih.gov/sites/entrez?Db=gene&Cmd=ShowDetailView&TermToSearch=22629) | tyrosine 3-monooxygenase/tryptophan 5-monooxygenase activation protein, eta polypeptide |
| 22628 | [*Ywhag*](http://www.ncbi.nlm.nih.gov/sites/entrez?Db=gene&Cmd=ShowDetailView&TermToSearch=22628) | tyrosine 3-monooxygenase/tryptophan 5-monooxygenase activation protein, gamma polypeptide |
| 22631 | [*Ywhaz*](http://www.ncbi.nlm.nih.gov/sites/entrez?Db=gene&Cmd=ShowDetailView&TermToSearch=22631) | tyrosine 3-monooxygenase/tryptophan 5-monooxygenase activation protein, zeta polypeptide |
| 67003 | [*Uqcrc2*](http://www.ncbi.nlm.nih.gov/sites/entrez?Db=gene&Cmd=ShowDetailView&TermToSearch=67003) | ubiquinol cytochrome c reductase core protein 2 |
| 22239 | [*Ugt8a*](http://www.ncbi.nlm.nih.gov/sites/entrez?Db=gene&Cmd=ShowDetailView&TermToSearch=22239) | UDP galactosyltransferase 8A |
| 13867 | [*Erbb3*](http://www.ncbi.nlm.nih.gov/sites/entrez?Db=gene&Cmd=ShowDetailView&TermToSearch=13867) | v-erb-b2 erythroblastic leukemia viral oncogene homolog 3 (avian) |
| 19697 | [*Rela*](http://www.ncbi.nlm.nih.gov/sites/entrez?Db=gene&Cmd=ShowDetailView&TermToSearch=19697) | v-rel reticuloendotheliosis viral oncogene homolog A (avian) |
| 20955 | [*Vamp7*](http://www.ncbi.nlm.nih.gov/sites/entrez?Db=gene&Cmd=ShowDetailView&TermToSearch=20955) | vesicle-associated membrane protein 7 |
| 22393 | [*Wfs1*](http://www.ncbi.nlm.nih.gov/sites/entrez?Db=gene&Cmd=ShowDetailView&TermToSearch=22393) | Wolfram syndrome 1 homolog (human) |
| 22433 | [*Xbp1*](http://www.ncbi.nlm.nih.gov/sites/entrez?Db=gene&Cmd=ShowDetailView&TermToSearch=22433) | X-box binding protein 1 |
| 22632 | [*Yy1*](http://www.ncbi.nlm.nih.gov/sites/entrez?Db=gene&Cmd=ShowDetailView&TermToSearch=22632) | YY1 transcription factor |
| 22771 | [*Zic1*](http://www.ncbi.nlm.nih.gov/sites/entrez?Db=gene&Cmd=ShowDetailView&TermToSearch=22771) | zinc finger protein of the cerebellum 1 |
| 27801 | [*Zdhhc8*](http://www.ncbi.nlm.nih.gov/sites/entrez?Db=gene&Cmd=ShowDetailView&TermToSearch=27801) | zinc finger, DHHC domain containing 8 |
